# Oncogenic signaling pathway dysregulation landscape reveals the role of pathways at multiple omics levels in pan-cancer

**DOI:** 10.3389/fgene.2022.916400

**Published:** 2022-08-17

**Authors:** Na Wang, Dan-Ni He, Zhe-Yu Wu, Xu Zhu, Xiao-Ling Wen, Xu-Hua Li, Yu Guo, Hong-Jiu Wang, Zhen-Zhen Wang

**Affiliations:** ^1^ Key Laboratory of Tropical Translational Medicine of Ministry of Education, College of Biomedical Information and Engineering, Hainan Medical University, Haikou, China; ^2^ College of Bioinformatics Science and Technology, Harbin Medical University, Harbin, China; ^3^ College of Biomedical Information and Engineering, Hainan Medical University, Haikou, China

**Keywords:** signaling pathways, dysregulation landscape, molecular subtypes, multi-omics, biomarkers, pan-cancer

## Abstract

Dysregulation of signaling pathways plays an essential role in cancer. However, there is not a comprehensive understanding on how oncogenic signaling pathways affect the occurrence and development with a common molecular mechanism of pan-cancer. Here, we investigated the oncogenic signaling pathway dysregulation by using multi-omics data on patients from TCGA from a pan-cancer perspective to identify commonalities across different cancer types. First, the pathway dysregulation profile was constructed by integrating typical oncogenic signaling pathways and the gene expression of TCGA samples, and four molecular subtypes with significant phenotypic and clinical differences induced by different oncogenic signaling pathways were identified: TGF-β+ subtype; cell cycle, MYC, and NF2− subtype; cell cycle and TP53+ subtype; and TGF-β and TP53− subtype. Patients in the TGF-β+ subtype have the best prognosis; meanwhile, the TGF-β+ subtype is associated with hypomethylation. Moreover, there is a higher level of immune cell infiltration but a slightly worse survival prognosis in the cell cycle, MYC, and NF2− subtype patients due to the effect of T-cell dysfunction. Then, the prognosis and subtype classifiers constructed by differential genes on a multi-omics level show great performance, indicating that these genes can be considered as biomarkers with potential therapeutic and prognostic significance for cancers. In summary, our study identified four oncogenic signaling pathway–driven patterns presented as molecular subtypes and their related potential prognostic biomarkers by integrating multiple omics data. Our discovery provides a perspective for understanding the role of oncogenic signaling pathways in pan-cancer.

## Introduction

A large number of studies have shown that the oncogenic signaling pathways play important roles in cancers, and multi-omics changes that occurred in these signaling pathways are identified as the common biomarkers in cancers. Therefore, the identification of oncogenic signaling pathways has become a key step in cancer drug screening and cancer treatment. Although the roles of individual pathways in the development of single cancer have been successively discovered and demonstrated, it is interesting to study how these signaling pathways affect cancer development and progression from a pan-cancer perspective.

There are many studies on oncogenic signaling pathways and the genes involved (Joerger and Fersht, 2016; Taciak et al., 2018; Calses et al., 2019). It has been reported that the RTK-RAS pathway, PI3K/Akt signaling pathway, TP53 signaling pathway, APC, and other signaling pathways often undergo genetic changes in cancer. Then, the molecular mechanism of these pathways and the role of each gene in these pathways and the relationship between these pathways and the occurrence and development of cancer were integrated (Vogelstein and Kinzler, 2004). Francisco used multi-omics data to analyze the mechanisms and patterns of 10 pathways, including cell cycle, Hippo, MYC, NOTCH, Nrf2, PI3Ki-Akt, RTK-RAS, TGF-β, p53, and β-catenin/WNT, and identified the interaction of pathways (Sanchez-Vega et al., 2018). The study has proven that the main functions of the Hippo pathway include restriction of tissue growth and regulation of cell proliferation, differentiation, and migration in developing organs. In addition, the dysregulation of the Hippo pathway can also lead to abnormal cell growth and the occurrence of tumors (Meng et al., 2016). Giachino et al. (2015) explored the role of the NOTCH signaling pathway in promoting and suppressing cancer and analyzed the molecular mechanisms of the NOTCH signaling pathway in hematological cancers and solid tumors, which have also been linked to therapeutic strategies targeting the NOTCH pathway in human cancer treatment.

In recent years, the research on subtype analysis of single cancer based on pathways has been continuously developed (Bild et al., 2006; Liu et al., 2015; Kaunitz et al., 2017; Thanki et al., 2017). Bidkhori et al. (2018) classified hepatocellular carcinoma (HCC) patients into three subtypes with significant differences based on graph and control theory concepts to the topology of genome-scale metabolic networks and identified drug targets for effective treatment of HCC patients.

Gong et al. (2021) discovered three subtypes of triple-negative breast cancer (TNBC) with significant prognosis, molecular subtype distribution, and genomic alterations by investigating metabolic pathways, which demonstrated the metabolic heterogeneity of TNBC and made it possible to develop personalized treatments for unique tumor metabolism characteristics. Park et al. (2019) identified glioblastoma multiforme (GBM) subtypes with prognostic core genes, prognostic chromosomal aberrations, and mutations. The aim was to verify that the failure of targeted therapy in patients with glioblastoma is associated with high heterogeneity and activation of multiple oncogenic pathways. It is believed that subtype-specific alterations can be used as new prognostic biomarkers and therapeutic targets for GBM. Moreover, although the pan-cancer analysis can open the doors to identification of the commonalities in cancer and offer insights that could expand further discoveries and cancer treatments, there are few studies focused on the dysregulated patterns of multiple signaling pathways systematically in pan-cancer, and the cooperative mode of oncogenic signaling pathways is not clear.

Here, we proposed a method to identify different roles of oncogenic signaling pathways from the perspective of pan-cancer. The four molecular subtypes named by different signaling pathways were identified based on the gene expression of TCGA data, which shows distinct phenotypic and clinical features. In addition, combining multi-omics data, we studied the differences in differentially expressed genes, copy number variations, chromatin accessibility, DNA methylation levels, and tumor microenvironment of the four subtypes, and identified differential genes of each omics which were used to construct the prognostic models with significant results, such as WNT7A, CNTN6, and CDR1. These differential signatures were characterized as biomarkers with potential therapeutic and prognostic significance for cancer. In conclusion, the research helps to further understand the role of oncogenic signaling pathways in pan-cancer.

## Results

### Four pathway-driven subtypes were identified based on oncogenic signaling pathways

**FIGURE 1:**
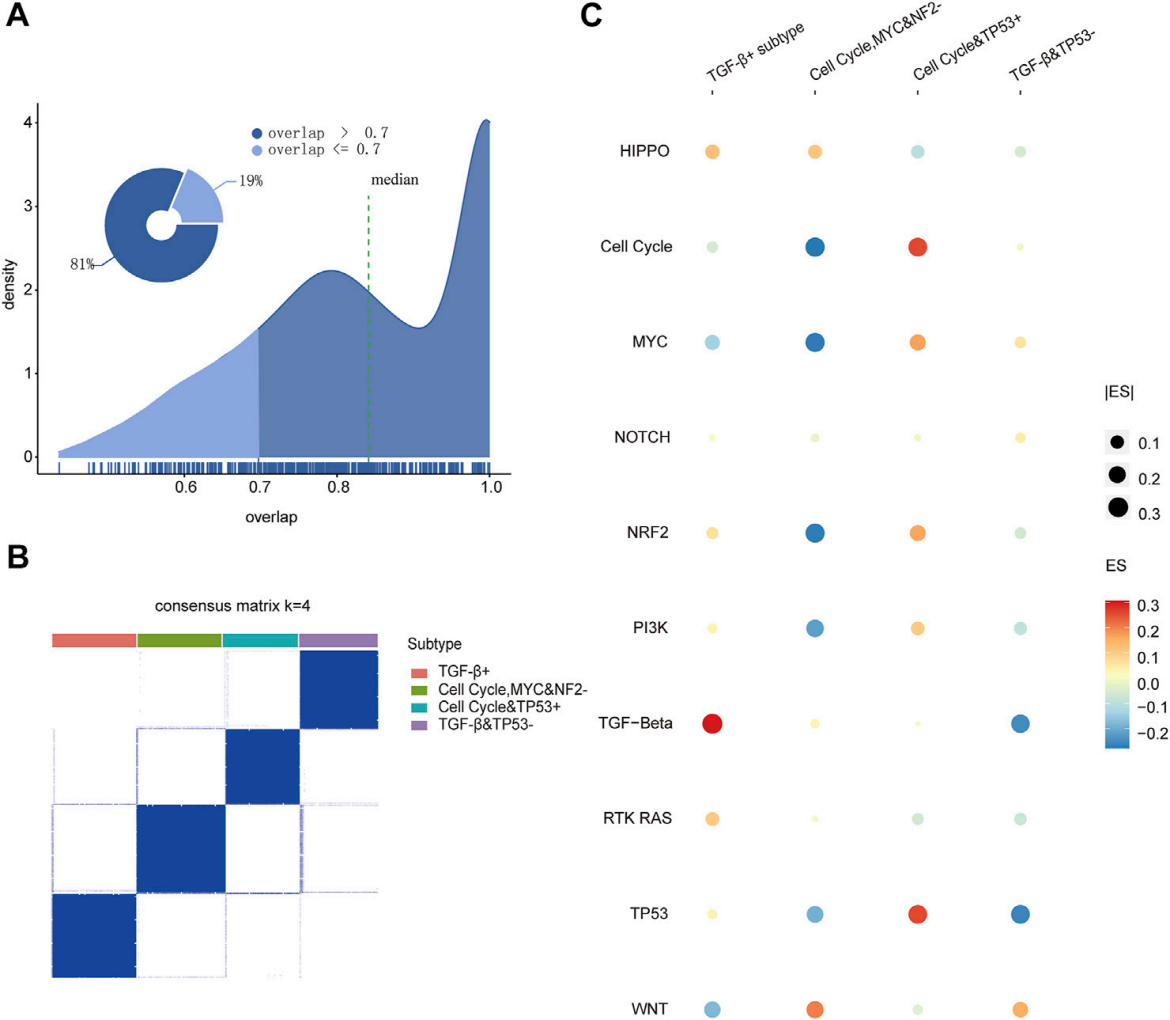
**(A)** Coincidence rate of the clustering results under different clustering methods and measures. **(B)** Consistent clustering result by the kmdist-Spearman method to cluster the TCGA training set into four classes. **(C)** Dot plot of the enrichment scores for 10 pathways in four subtypes.

In order to investigate the mechanism of 10 pathways in cancers (Ciriello et al., 2013; Imperial et al., 2019; Paczkowska et al., 2020), we collected 333 genes of 10 canonical oncogenic signaling pathways confirmed in the previous research. Based on those gene expression levels for 7,518 patients (TCGA training set, Supplementary Table S1), we first characterized the oncogenic signaling pathway dysregulation landscape by calculating the enrichment scores of 10 pathways for each patient with the GSVA package in R (Supplementary Figure S1D) (Hanzelmann et al., 2013), and then using the consensus cluster analysis (Wilkerson and Hayes, 2010; Gan et al., 2018), we identified distinct clusters with the oncogenic signaling pathway dysregulation landscape. To get the more robust clustering results, the consistency of the clustering results was evaluated between different cluster methods and measurements. There were about 81% of the clustering results whose consistency rate reached 0.7 in all the cluster results. It showed that the clustering results were consistent under different clustering methods and measurements, which suggests that there are significant different subtype patient groups in pan-cancer (Figure 1A). Then, the consensus clustering results when k = 2–8 were discussed (Supplementary Figure S1). The variation trend of the area under cumulative density function curve (CDF) is shown in Supplementary Figure S1B, and the result at k = 4 was the inflection point in all outcomes. Under k = 4, we observed the clarity of classification of clustering results among 112 clustering results, and then, the result with the measurement of the kmdist-Spearman method was considered as the final result of clustering (Figure 1B), which indicates that four robust consensus molecular subtypes driven by specific oncogenic signaling pathways were identified. The heat map shows the enrichment score profile of 10 pathways for four molecular subtypes in Figure 1C; it exhibits that the TGF-β pathway is upregulated in subtype 1, then cell cycle, MYC and NF2 pathways are downregulated in subtype 2, while subtype 3 is basically opposite to subtype 2, and the TGF-β and TP53 pathways are downregulated in subtype 4. Therefore, we named the four molecular subtypes based on the characteristics of being driven by the pathways as the TGF-β+ subtype (subtype 1); cell cycle, MYC, and NF2− subtype (subtype 2); cell cycle and TP53 + subtype (subtype 3); and TGF-β and TP53− subtype (subtype 4), respectively.

### Four subtypes based on oncogenic signaling pathways show phenotypic and clinical heterogeneity

**FIGURE 2:**
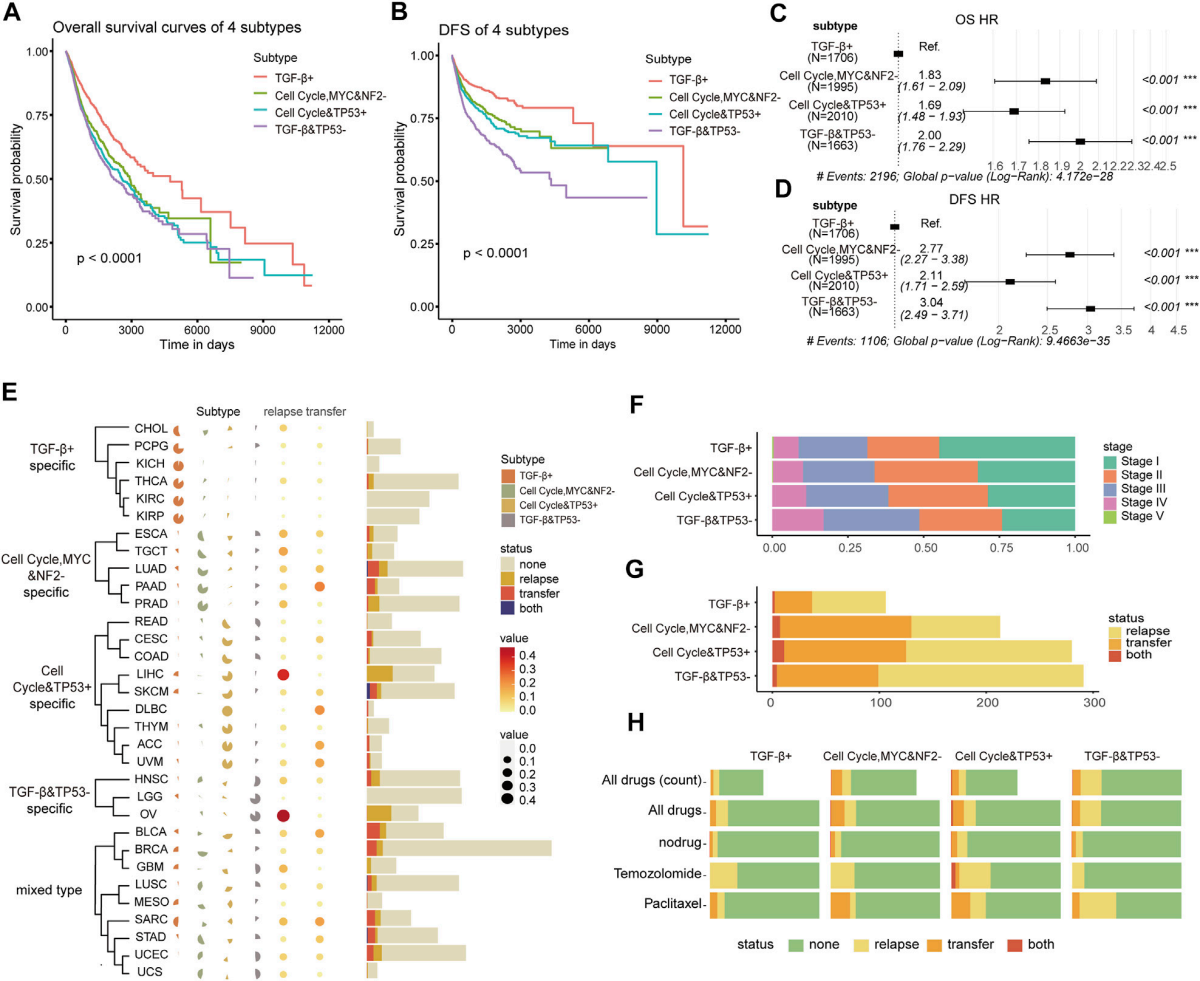
**(A,B)** Kaplan–Meier curves of the overall survival (OS) and disease-free survival (DFS) among the four subtypes in TCGA training cohort. **(C,D)** Forest plot of single Cox regression analysis on subtypes for OS and DFS. The hazard ratios are shown with 95% confidence intervals. **(E)** Percentage heat map shows the distribution of 32 cancers in four subtypes (left), and the dotted heat map and histogram show the distribution of 32 cancers in the recurrence and metastasis state (right). **(F)** Distribution of the four subtypes in the pathological stage. **(G)** Distribution of the four subtypes in the recurrence and metastasis state. **(H)** Sensitivity to drugs of four subtypes’ patients. (None: sensitive; relapse, transfer, both: insensitive).

To explore if there is the phenotypic and clinical heterogeneity among those oncogenic signaling pathway–driven molecular subtypes, we first continued to compare the survival differences among patients in various molecular subtypes using the Kaplan–Meier curve and the log-rank test (Xie and Liu, 2005). It represents significant differences in overall survival and disease-free survival time among the patients of the four subtypes. The TGF-β+ subtype had significantly better overall survival (OS) and disease-free survival (DFS) (OS: Log rank, *p* < 0.0001, Figure 2A; DFS: Log rank, *p* < 0.0001, Figure 2B) than other subtypes. To investigate whether these results hold for a specific cancer type or were only valid to “pan-cancer”, we ran an analysis of the differences in survival curves across the four subtypes within each cancer type. It showed that the results of the survival curves remained similar when compiling everything into pan-cancer; there were differences only in CHOL, COAD, and THCA, but it was considered due to the small sample size of the subtype (Supplementary Figure S3).

A Cox hazard regression analysis was used to compare the hazard ratio of OS and DFS among the four subtypes. Using the TGF-β+ subtype as the reference group, we found that the other three subtypes were significantly at a high risk for both OS and DFS, suggesting that there was a relationship between poor prognosis and the molecular subtypes driven by oncogenic signaling pathways (Figures 2C,D). The results showed that the hazard ratio of the TGF-β+ subtype was different from the other three subtypes, indicating that the subtype characteristics were independent predictors of patient survival. The multiple Cox regression analysis also revealed that the pathological stage was a risk factor for poor prognosis (Supplementary Figures S2A,C). Then, we investigated if age and sex contributed to the different hazard ratios among these subtypes and found out that age > 60 was an important high risk factor for survival both in OS and DFS, but the sex information contributed to the hazard ratio only in OS (Supplementary Figures S2B,D).

Next, we analyzed the distribution of cancer types among patients to find out whether a cancer type is specifically enriched in these subtypes. Cancers in the kidney with relatively better prognosis are mainly enriched in the TGF-β+ subtype, intestinal cancers are predominant in cell cycle and TP53 + subtype-specific, and head and neck cancers are enriched in TGF-β and TP53− subtype. This demonstrated that the distribution of cancer types in the molecular subtypes may be tendentious, so we categorized cancer types by molecular subtypes to understand whether cancer type specific to the same subtype tend to be driven by the same pathways, leading to similar mechanisms of cancer pathogenesis. The TGF-β+ subtype was significantly enriched in CHOL, PCPG, KICH, THCA, KIRC, and KIRP; THE cell cycle, MYC, and NF2− subtype was significantly enriched in ESCA, TGCT, LUAD, PAAD and PRAD, and the cell cycle the TP53 + subtype was significantly enriched in READ, CESC, COAD, LIHC, SKCM, DLBC, THYM, ACC, and UVM; the TGF-β and TP53− subtype was significantly enriched in HNSC, LGG, and OV. Nonetheless, the other nine mixed cancer types of BLCA, BRCA, GBM, LUSC, MESO, SARC, STAD, UCEC, and UCS were classified as mixed carcinomas, and there was no significant difference enrichment among those subtypes (Figure 2E). Furthermore, we continued to check if the patients of cancer types enriched in the subtypes with poor prognosis tend to metastasis or recurrence. The proportion of recurrence and metastasis of the patients in CHOL, PCPG, KICH, THCA, KIRC, and KIRP enriched in the TGF-β+ subtype were significantly lower than those of other cancers, and the recurrence rate of the cell cycle, MYC and NF2 subtype-specific patients was significantly higher than that of metastasis. Most patients with cell cycle and TP53+ subtype-specific cancers were more likely to develop metastases than local recurrence. There was no significant difference in recurrence and metastasis of mixed carcinomas. In other words, four oncogenic pathway–related subtypes have tissue specificity and are closely related to the recurrence and metastasis.

We further explored the reasons for differences in patient survival and analyzed the pathological stage distribution of patients among the four subtypes. From the TGF-β+ subtype to TGF-β and TP53− subtype, the proportion of patients in the early stage gradually decreased and the proportion in the late stage gradually increased, which was consistent with the survival analysis, indicating that the four subtypes’ patients have significant differences in pathological stages (Figure 2F). At the same time, the patients of the four subtypes also showed differences in recurrence and metastasis rates. The patients of the TGF-β+ subtype with the best prognosis owned the lowest rate of recurrence and metastasis, while the patients of the TGF-β and TP53− subtype with the worst prognosis owned a lower metastasis rate than the patients of the cell cycle, MYC, and NF2− subtype and cell cycle and TP53 + subtype, but it had a significantly higher recurrence rate (Figure 2G), indicating that those subtypes’ patients owned specific pathogenic molecular mechanisms which determined the postoperative pathological stage of the patient. Then, we used Fisher’s test to analyze the status of recurrence and metastasis of patients after drug treatment in four subtypes. First, we screened out the drugs which were used by more than 50 patients for analysis (Supplementary Figure S4A). The patients of the TGF-β+ subtype showed the smallest proportion of recurrence or metastasis after drug treatment. Temozolomide was significantly less sensitive in cell cycle and TP53 + subtype patients (*p* < 0.05), and paclitaxel was significantly less responsive in TGF-β and TP53− subtype patients (Figure 2H; Supplementary Figure S4B). It means that temozolomide may be related to the upregulation of the activity of the cell cycle and the TP53 pathway and is also effective for the diseases caused by the dysregulation of these two pathways.

Collectively, the patients of four subtypes based on oncogenic signaling pathways had significant differences in clinical phenotypes, such as survival time, tissue specificity, tumor stage, recurrence and metastasis rates, and drug response. The patients with the upregulated TGF-β pathway had the best prognosis, while patients with downregulated TGF-β and TP53 pathways had the worst prognosis. These data imply that the pathogenesis of cancer is strongly correlated with the molecular mechanisms of oncogenic signaling pathways, and the dysregulation of pathways might be the driving factor for cancer development.

### Novel subtype and prognostic classifiers were constructed based on the genes related to prognosis among subtypes

**FIGURE 3:**
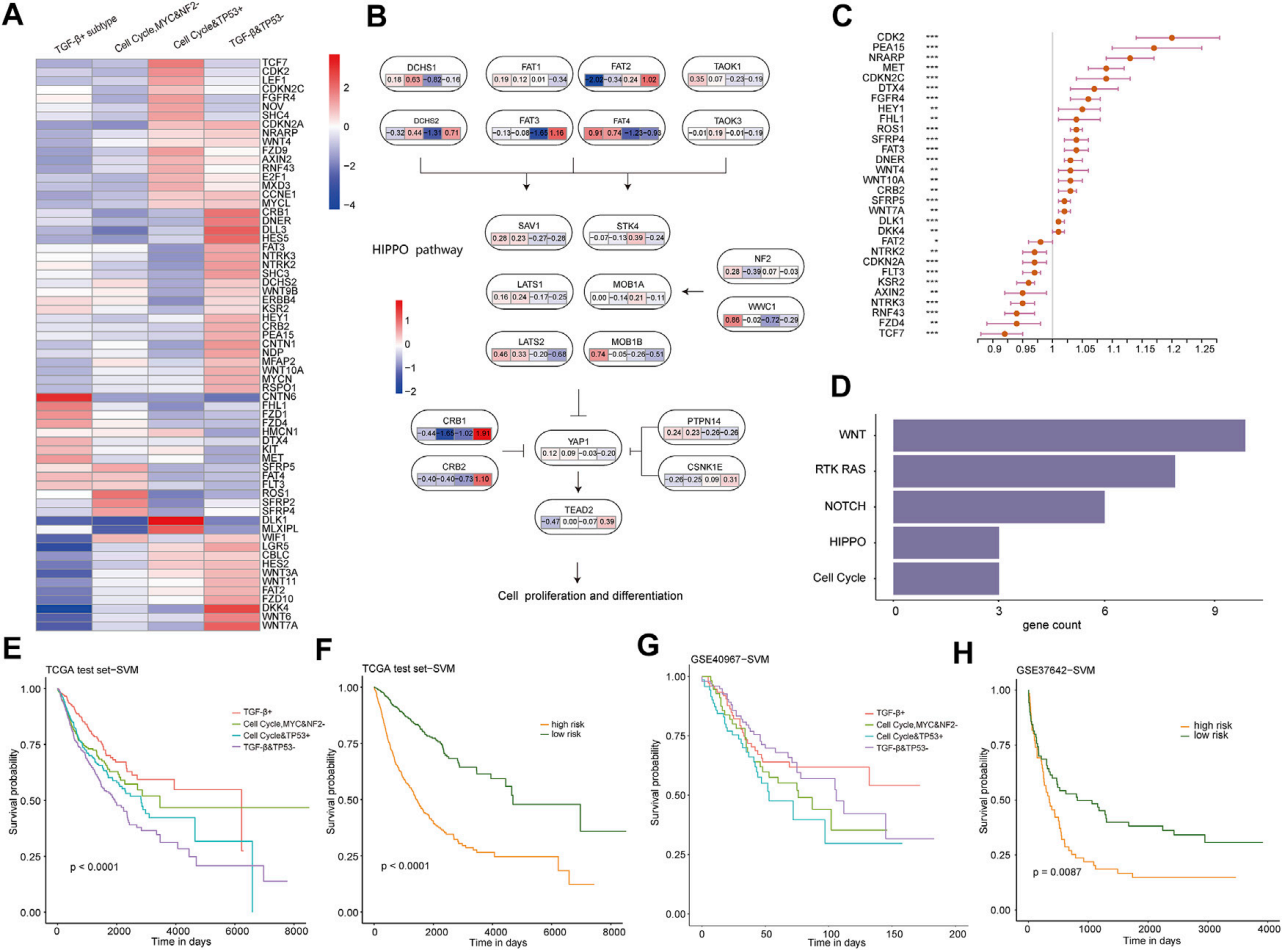
**(A)** Heat map of the log2FC value of differentially expressed genes in four subtypes. (FC, fold change, the ratio of the average mRNA expression for each cancer pathway–related subtype to the average mRNA expression for samples not of the aforementione subtype. Red, upregulated; blue, downregulated.). **(B)** Interaction of genes in the HIPPO pathway and the FC value of the four subtypes of the gene. **(C)** Forest plot of multivariate Cox regression analysis for 30 genes related to prognosis. The hazard ratios are shown with 95% confidence intervals (***p < 0.001; **p < 0.01; *p < 0.05; and *p* < 0.1). **(D)** Pathways in which 30 differentially expressed genes are enriched. **(E)** KM survival curves of the subtype classifier constructed using samples from TCGA test cohort by the SVM method. **(F)** KM survival curves of the prognosis classifier constructed using samples from TCGA test cohort by the SVM method. **(G)** KM survival curves of the subtype classifier constructed using GSE40967 by the SVM method. **(H)** KM survival curves of the prognosis classifier constructed using GSE37642 by the SVM method.

To figure out whether transcriptional changes among subtypes are related to the dysregulation of specific signaling pathways, we estimated the gene expression difference in these pathways in TCGA training cohort. According to 333 cancer-related pathway gene expressions, 65 differentially expressed genes between each subtype and other subtypes were identified (*p* < 0.05 and fold change | log2FC | > 1, Supplementary Table S2) (Robinson et al., 2010). Most genes showed high expression in the cell cycle and TP53 + subtype and the TGF-β and TP53− subtype, and just a few genes showed high expression in the TGF-β+ subtype and the cell cycle, MYC, and NF2− subtype (Figure 3A). Thereafter, by mapping differentially expressed genes into these oncogenic signaling pathways, some subtype-specific key sub-pathways with consistent transcriptional change were identified (Figure 3B; Supplementary Figures S5–S7). For example, NF2 and WWC1 were highly expressed in the TGF-β+ subtype, which promotes the high expressions of LATS1, SAV1, and other genes in the TGF-β+ subtype, whereas CRB1 and CRB2 were highly expressed in the TGF-β and TP53− subtype, inhibiting the *YAP1* gene, making it lowly expressed in the TGF-β and TP53− subtype in the HIPPO pathway. This result showed that the different driver genes might lead to the different pathway changes in the TGF-β+ subtype and the TGF-β and TP53− subtype, which suggests that the oncogenic signaling pathways own subtype-specific driving sub-pathways, resulting in different states of dysregulation of downstream pathways.

Furthermore, we explored whether these 65 differentially expressed genes would predict a worse prognosis in pan-cancer. A total of 56 prognostic-related genes were identified by using a single Cox regression analysis, and multivariate Cox proportional hazard models revealed 30 genes which can predict worse prognosis (Figure 3C). These 30 genes were enriched into WNT, RTK-RAS, NOTCH, HIPPO, and cell cycle pathways (Figure 3D). In particular, there are 10 differentially expressed genes associated with prognosis enriched in the WNT pathway, which might be part of the reason for the upregulation of the WNT pathway activity in the cell cycle, MYC, and NF2− subtype and TGF-β and TP53− subtype. Collectively, our results demonstrate that there are strong relationships between pathway dysregulation and the subtypes. We further explored to construct a subtype and prognostic classifiers, based on the expression profiles of these 30 genes, by using the support vector machine (SVM) method. Then, TCGA test cohort was used to verify these 30 genes as biomarkers for predicting subtype and prognosis, and the classifiers’ results in survival were also very significant (Figures 3E,F, *p* < 0.0001). At the same time, GSE40967 and GSE37642 data on the GPL570 platform from the Gene Expression Omnibus (GEO) database were downloaded as validating (external verification) data sets. Then, SVM was used to build the classifiers, and the classification result had significant survival differences between our prognostic subgroups (Figure 3H). The Kaplan–Meier curve of GSE40967 data also revealed distinct prognostic outcomes among the predicted subtypes, although the difference was not statistically significant (*p* = 0.08, Figure 3G), possibly due to the single cancer type included in the data.

These results suggest that these 30 differentially expressed genes associated with prognosis among subtypes could be recognized as key genes in oncogenic signaling pathways and biomarkers for identifying molecular subtypes and risk groups, and their expression changes can also affect the expression of upstream and downstream genes through the relationship of promotion or inhibition between genes, leading to dysregulation of oncogenic signaling pathways.

### Oncogenic signaling pathway–based subtypes show distinct genomic alteration features

**FIGURE 4:**
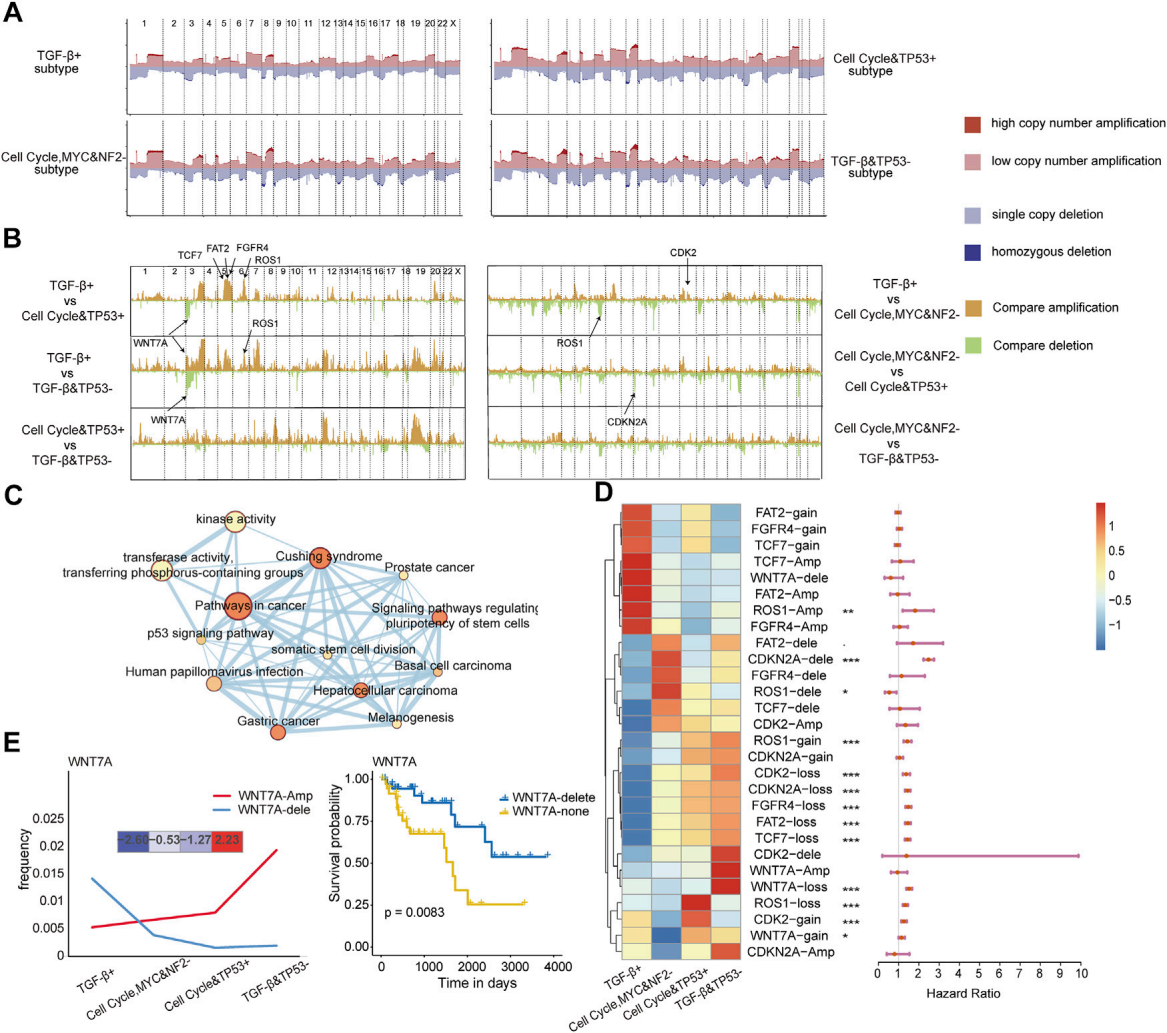
**(A)** Somatic CNA frequency of individual genes in each subtype plotted along the chromosomes. **(B)** Comparisons of somatic CNA between subtypes with −log10 FDR plotted along the chromosomes (Fisher’s exact test). **(C)** Interaction of the enriched pathways. The size represents the number of genes, and the color represents the *p*-value. **(D)** Differences in copy number variation across the four subtypes of the four copy number variation states of the seven genes and their relationship with the prognosis. **(E)** Changes in the number of amplified and deleted samples of WNT7A in the four subtypes; the expression of WNT7A in the four subtypes (left) and the difference in survival between the two categories (right).

Genomic alterations can drive oncogenic signaling pathway reprogramming in cancers. We further explored to compare genomic alterations among the four subtypes with the copy number variation data on 22,445 genes obtained from UCSC Xena for TCGA pan-cancer patients. Genome-wide copy number variation revealed that the TGF-β and TP53− subtype had a significantly higher copy number variation, especially on chromosomes 3, 4, and 19, as shown in Figure 4A. We further examined the detailed characterization of copy number variation across the subtypes. Between any two subtypes, the differences of all genes in copy number amplification and deletion (−log10 FDR value) were calculated using Fisher’s exact test (Figure 4B). There were significant difference peaks on chromosomes 3, 5, and 6 between the TGF-β+ subtype and the cell cycle and TP53+ subtype; on chromosomes 3, 5, 7, and 19 chromosomes between the TGF-β+ subtype and the TGF-β and TP53− subtype; and on chromosomes 12 and 19 between the cell cycle and TP53 + subtype and the TGF-β and TP53− subtype. Combined with pathway-related genes, especially in chromosome 3, we found that there were seven genes, namely, *FAT2*, *CDK2*, *CDKN2A*, *WNT7A*, *TCF7*, *FGFR4*, and *ROS1*(−log10 FDR>2), which had significant differences in the copy number between subtypes.

To further explore the biological functions of these seven genes, we performed a pathway enrichment analysis for these genes. In addition to affecting oncogenic pathways, we further examined the biological functions of these genes to see if they affect cancer development from other perspectives. The results showed that the seven genes were also enriched in the pathways, including Cushing syndrome, and the pathways of cancer and kinase activity (Figure 4C). These genes were indeed involved in cancer development as a multifunctional model, and this result suggests that the genomic alterations of these genes may drive the dysregulation of oncogenic signaling pathways. Then, the copy number variation states of the seven genes which had different copy number changes between subtypes were disassembled to analyze. We found that FAT2-amp, FGFR4-amp, TCF7-amp, and WNT7A-delete showed upregulation in the TGF-β+ subtype, and most other genes showed upregulation in the other three subtypes. Multivariate Cox proportional hazard models also revealed the prognosis-related states in non-diploid normal copy states of the seven genes (Figure 4D). The amplification frequency of WNT7A gradually increased from the TGF-β+ subtype to the TGF-β and TP53− subtype, and the frequency of WNT7A deletion gradually decreased from the TGF-β+ subtype to the TGF-β and TP53− subtype. The most deleted changes and the least amplification changes of WNT7A in copy number variation were observed in the TGF-β+ subtype, which was similar to the WNT7A gene expression trend among the four subtypes. It shows that the copy number variation change of WNT7A affects its expression on the transcriptome and thereby affects the function of the WNT pathway, and this result suggests that the copy number variation of WNT7A could be a driver factor for WNT pathway dysregulation. Then, we continued to select the four copy number variation states of WNT7A as biomarkers for diagnosis. Notably, the survival of patients with homozygous deletion of WNT7A was significantly better than that of patients with normal diploid copies of WNT7A (*p* = 0.0083, Figure 4E), which validates the efficacy of WNT7A as a prognostic marker.

### Five subtype-specific enhancers were identified by a chromatin accessibility analysis

**FIGURE 5:**
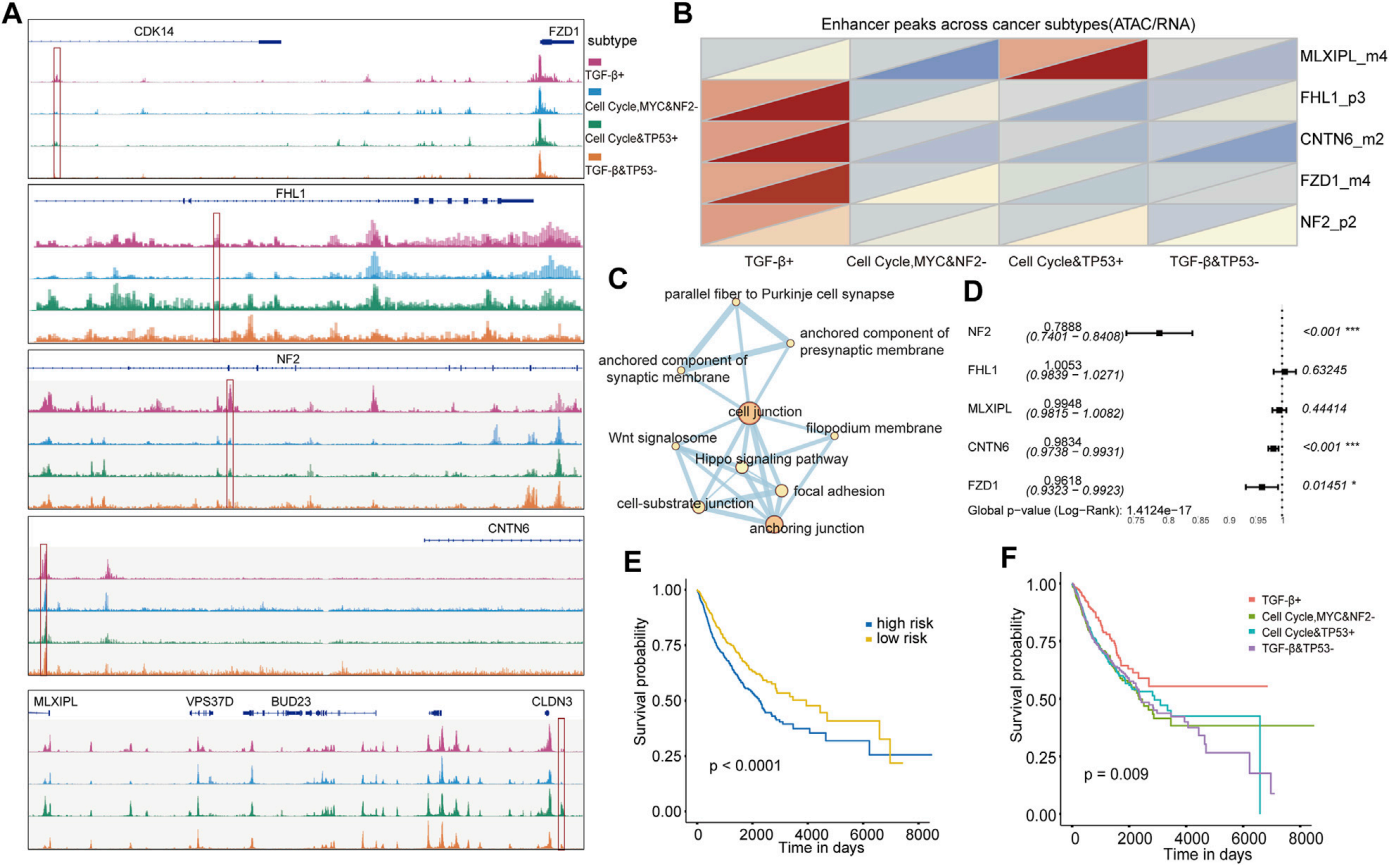
**(A)** Five enhancers visualized using IGV. **(B)** ATAC accessibility (upper triangle) and expression (lower triangle) heat map of five enhancer-related genes in four subtypes. **(C)** Pathways in which five enhancers are enriched. **(D)** Multi-Cox risk regression model of five enhancers. **(E)** KM survival curve for classifying the high and low risks of TCGA validation set samples was constructed by five enhancers as features. **(F)** KM survival curve of TCGA validation set samples divided into four subtypes by the classifier was constructed with five enhancers as features.

The integration of transcriptome data and ATAC-seq could determine a great deal of putative distal enhancers (Corces et al., 2018). We continued to identify subtype-specific transcriptional regulators that influence patterns of oncogenic pathway dysregulation at the level of chromatin accessibility by integrating ATAC-seq data with RNA-seq data for pan-cancer cases in TCGA. A total of 2,579 differential ATAC peaks between any subtypes were identified, and we found that there were five enhancers showing subtype-specific activity in the oncogenic signaling pathways such as CNTN6 in the NOTCH pathway and MLXIPL in the MYC pathway. Furthermore, a location analysis of these peaks showed that these subtype-specific enhancers’ chromosome locations were distinct. For example, FZD1_m4 and CNTN6_m2 were located in the distal upstream of the related genes, FHL1_p3 and NF2_p2 were located in the inner gene, and MLXIPL_m4 was located in the distal downstream of related genes (Figure 5A). We further investigated whether these enhancers located in different chromosomal regions could lead to its expression change. Subsequently, expression of these subtype-specific enhancer-related genes was analyzed, and it was found that the changes in chromosome accessibility and gene expression showed a consistent trend (Figure 5B). For example, MLXIPL displayed high chromatin accessibility and gene expression level in the cell cycle and TP53 + subtype, whereas it showed the opposite trend in the cell cycle, MYC, and NF2− subtype. This result suggests that these subtypes own their specific transcriptional regulators, which drive oncogenic signaling pathway dysregulation by distinct molecular mechanisms.

To understand the molecular function of the enhancers, we performed the functional enrichment analysis and found that the five genes were enriched in several other pathways, including cell junction and anchoring junction pathways (Figure 5C). The junctions of these pathways might affect cancer cell adhesion and further affect the possibility of metastasis. We further explored whether these subtype-specific enhancers could be used to predict a worse OS and construct subtype classifiers. NF2, CNTN6, and FZD1 presented very low risk. It suggests that the genes of *NF2*, *CNTN6*, and *FZD1* might be low risk factors for poor prognosis (Figure 5D). Using these five genes as features, we constructed subtype and prognostic classifiers using the random forest method. The patients were divided into high- and low-risk groups with significant survival differences according to the prognostic model risk score (*p* < 0.0001, Figure 5E). Also, the survival differences were also significant for subtype classifiers (*p* = 0.009, Figure 5F). Overall, our analysis revealed that these five genes can serve as key biomarkers for identifying patient prognostic risk and subtypes based on oncogenic signaling pathways.

### Pathway-driven subtype-associated methylation sites were identified

**FIGURE 6:**
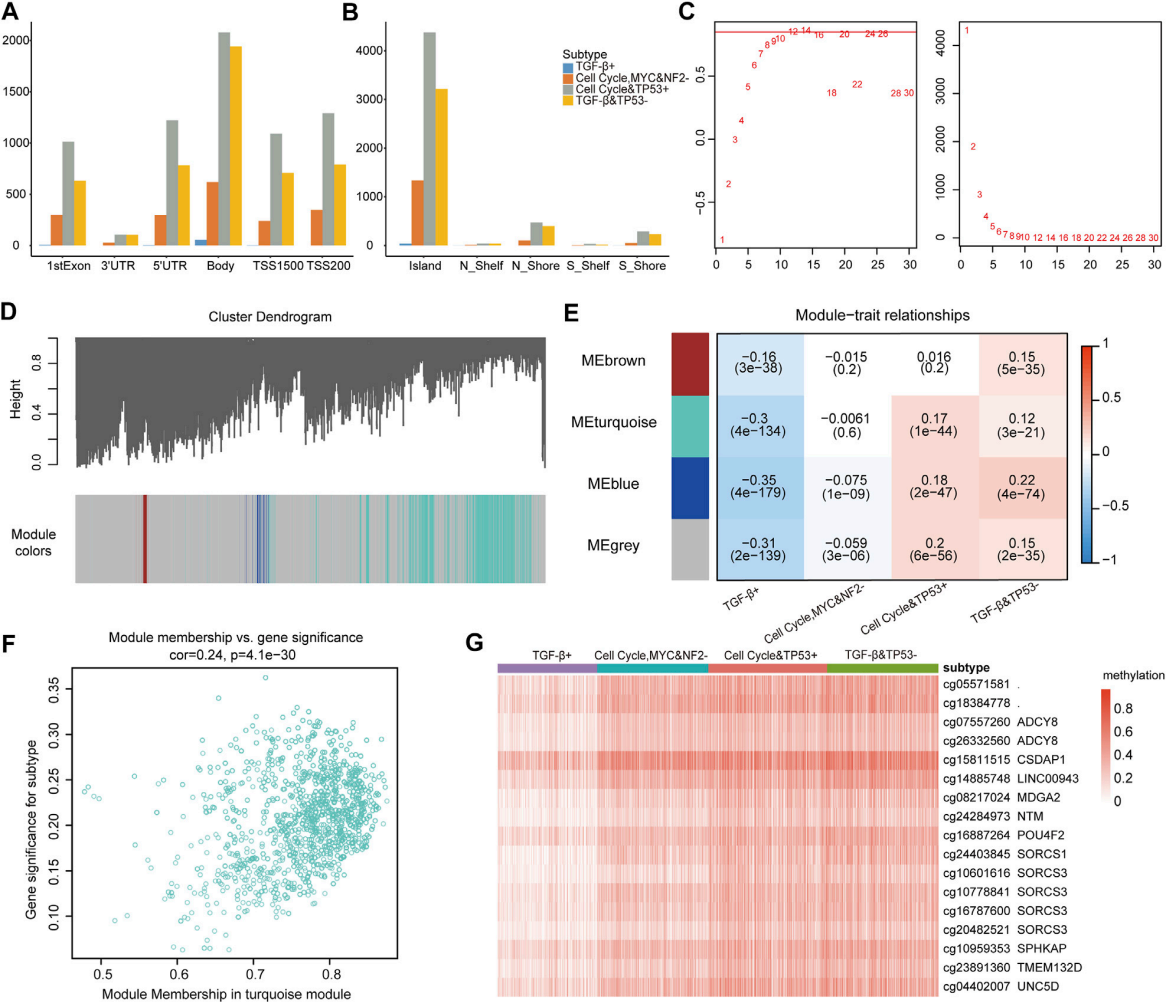
**(A)** Location of methylation sites on genes. **(B)** Location of methylation sites on CPG islands. **(C)** Analysis of network topology for different soft thresholding powers. **(D)** Module colors and gene dendrogram. **(E)** Correlations between the four modules and the subtype characteristics of samples. **(F)** Weighted interaction gene network of the CpG sites in the turquoise module. **(G)** Methylation profiles of the 15 CpG sites.

In tumor cells, proto-oncogenes are in a state of hypomethylation and activated, while tumor suppressor genes are in a state of hypermethylation and inhibited (Kulis and Esteller, 2010; Gyorffy et al., 2016; Chen et al., 2021). Next, we explored whether some methylated CPG sites had DNA methylation abnormalities due to subtypes driven by the oncogenic signaling pathway. We further performed a methylated CPG site analysis, and 11,122 differential methylated sites were identified. According to the methylation sites’ position on the gene, the differential methylation site of each subtype was classified (Figures 6A,B). There were the least differential methylated sites in 3′UTR, and most of the differential methylated sites were located on CpG islands. There were a few differential methylated sites in the TGF-β+ subtype, but much more in the cell cycle and TP53 + subtype and the TGF-β and TP53− subtype.

To compare methylation sites’ difference across subtypes, we further used the weighted gene co-expression network (WGCNA) (Langfelder and Horvath, 2008) to explore the subtype-specific driving methylation sites from the 11,122 methylation sites. After screening, the soft thresholding power of the WGCNA was 12 (Figure 6C). The network was constructed to classify all methylation sites into four modules (gray, brown, turquoise, and blue, Figure 6D). The correlations between the four modules and the subtype characteristics were obtained by using the phenotypic data on the patients (Figure 6E). It can be seen that methylation sites in the turquoise module are not only related to the turquoise module but also to its corresponding traits (Figure 6F), which further indicates that these sites are worthy of in-depth exploration.

We continued to identify subtypes related to the methylation sites, whose correlation with the turquoise module was greater than 0.8 and the subtype with a correlation greater than 0.25, and it revealed the strong correlations among these methylation sites. A total of 15 genes mapped by the DMSs (differentially methylated sites with a degree greater than 300) were identified (Supplementary Table S3). Furthermore, we estimated the methylation level of genes across subtypes. The methylation level of patients from the TGF-β+ subtype was significantly lower than that of patients from other subtypes, and the patients from the TGF-β+ subtype also had better prognosis than patients from other subtypes (Figure 6G). Then, these genes were mainly enriched in WNT, NOTCH, and RTK-RAS pathways. The results indicate that oncogenic signaling pathway–based subtypes are closely related to the methylation status, and the genes annotated at these 15 CpG sites are closely related to the dysregulation of oncogenic signaling pathways; also, hypomethylation is associated with a better prognosis for patients.

### Identification of tumor microenvironment–associated immune biomarkers across subtypes

**FIGURE 7:**
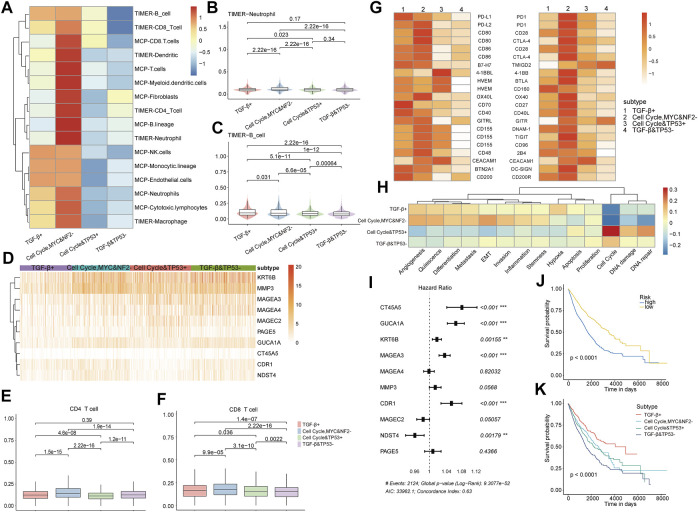
**(A)** Normalized infiltration of immune cells from TIMER and MCP in the four subtypes. **(B**,**C)** Violin chart of TIMER neutrophils and B-cell infiltration. **(D)** Gene expression heat map of subtype-specific T-cell dysfunction genes. **(E**,**F)** Boxplot of TIMER CD4 T-cell and CD8 T-cell infiltration. **(G)** Immune checkpoint tumor cells (left) and immune checkpoint T cells’ (right) corresponding gene normalized expression in the four subtypes. **(H)** Cell state enrichment scores’ heat map. **(I)** HR values of 10 subtype-specific T-cell dysfunction genes. **(J**,**K)** KM survival curves of the prognostic classifier (left) and subtype classifier (right) constructed with 10 subtype-specific T-cell dysfunction genes as features.

The tumor microenvironment (TME), the environment for tumor cells to survive, could facilitate tumor cell growth, metastasis, and immune escape. We estimated whether these oncogenic signaling pathway–based subtypes would show distinct tumor microenvironment characteristics. We first analyzed the infiltration level of immune cells estimated by TIMER and MCP of the four subtypes’ patients and found these subtype patients with specific tumor microenvironments. It was mainly reflected in the fact that the infiltration of most immune cells in the cell cycle, MYC, and NF2− subtype was significantly higher than that of other subtypes (Figure 7A, Supplementary Figure S8), especially neutrophils (Figure 7B) and B cells (Figure 7C), whereas we found that patients of the cell cycle, MYC, and NF2− subtype had a higher level of immune cell infiltration but a poor prognosis. Therefore, we continued to analyze this issue from the perspective of immune cell function such as T-cell dysfunction (Jiang et al., 2018; Zhao et al., 2020) and immune checkpoints. A total of 10 differentially expressed T-cell dysfunction–related genes were identified across subtypes, and these genes all showed significantly high expression in the cell cycle, MYC, and NF2− subtype (Figures 7D–F), which suggested that most patients in the cell cycle, MYC, and NF2− subtype exhibited a state of T-cell dysfunction. We continued to check the immune checkpoint genes’ expression level across subtypes and found that immune checkpoint genes also tended to be highly expressed in the cell cycle, MYC, and NF2− subtype (Figure 7G). Immune checkpoint genes were overexpressed, which can lead to suppressed immune function and cause low body immune capacity. In general, our analysis suggests that high gene expression of T-cell dysfunction and immune checkpoint genes might be responsible for the patients owning a higher level of immune cell infiltration but a lower prognosis in the cell cycle, MYC, and NF2− subtype. Next, we analyzed 14 cell states of the four subtypes’ patients based on the gene set variation analysis (GSVA) (Yuan et al., 2019). Most cell states except the cell cycle showed upregulation in the TGF-β+ subtype and the cell cycle, MYC, and NF2− subtype, and cell cycle, DNA damage, and DNA repair showed upregulation in the cell cycle and TP53 + subtype (Figure 7H). Overall, the aforementioned results reveal significant differences in immune cell infiltration, T-cell function, and cell state across subtypes.

Next, we continued to analyze whether the aforementioned 10 T-cell dysfunction gene expression models could predict patient prognosis and subtype. There were five genes with significantly high risk, and only one gene showed significantly low risk (Figure 7I). These genes were identified as key prognostic factors and then used as features to construct prognosis and subtype classifiers; both classifiers showed great performance (KM survival curve, log rank: *p* < 0.0001, Figures 7J,K).

## Materials and methods

### TCGA data sets

The gene expression data on 32 cancers including 9,398 samples were downloaded from UCSC Xena (https://xenabrowser.net/), and the data types were mRNA count-UQ and mRNA FPKM-UQ. We divided all TCGA patients into the training data set (80%) and the test data set (20%).

Then, the copy number variation data on 22,445 genes were obtained from UCSC Xena. The copy number variation data on TCGA samples included the four non-diploid normal copy states of homozygous deletion (−2), single copy deletion (−1), low copy number amplification (1), and high copy number amplification (2).

The clinical data on TCGA samples including gender, age, tumor weight, TNM stage, and survival time were downloaded by the GDC tool (https://portal.gdc.cancer.gov/).

### Gene expression omnibus data sets

We downloaded GSE40967 and GSE37642 data on the GPL570 platform from the Gene Expression Omnibus (GEO) database as an external validation data set. (https://www.ncbi.nlm.nih.gov/geo/). GSE40967 contained two sets of data, GSE39582 had 585 tumor samples including 566 CC samples and 19 non-tumor samples. GSE40966 had 566 tumor samples. The data contained clinical information including sex, age, TNM stage, treatment strategy, survival time, and mutation information. GSE37642 contained the expression data on 562 samples of adult acute myeloid leukemia (AML) patients. The clinical information included age and survival status.

### ATAC-seq data set

The genome-wide chromatin accessibility profiles (Corces et al., 2018) of 410 tumor samples spanning 23 cancer types from TCGA were downloaded by the GDC tool (https://portal.gdc.cancer.gov/).

### Immune cells

The tumor purity of the six immune cells, namely, B cells, CD4+ T cells, CD8+ T cells, neutrophils, macrophages, and dendritic cells of TCGA cancer patients, were available from TIMER (version 1.0) (Li et al., 2017) (http://cistrome.dfci.harvard.edu/TIMER/).

### DNA methylation data

The DNA methylation 450 k data on 31 cancers were downloaded from UCSC Xena. The data recorded the DNA methylation value (β value) of each array probe in each sample. The DNA methylation value is a continuous variable between 0 and 1, which represents the degree of methylation. A higher β value represents hypermethylation, and a lower β value represents hypomethylation.

We used the Xena probeMap derived from GEO GPL13534 to map the microarray probes to the coordinates of the human genome, displaying the annotation information of all methylation sites, including base changes, chromosomes, CPGs, and gene positions.

### Gene set variation analysis to calculate the enrichment score of each pathway

Gene set variation analysis (GSVA) (Hanzelmann et al., 2013) is a non-parametric, unsupervised method that estimates the enrichment score of each gene set based on the gene expression level. We used the R package “GSVA” (version 1.38.2) to calculate the enrichment scores of 10 oncogenic signaling pathways for each sample and built a pathway dysregulation profile. In the profile, the enrichment score greater than 0 means that the pathway activity is upregulated, while an enrichment score less than 0 indicates that the pathway activity is downregulated. The enrichment score is close to 0, which means that there is little difference in the pathway activity (http://www.bioconductor.org/packages/release/bioc/html/GSVA.html).

### Consensus cluster on training samples

We used the ConsensusClusterPlus package (version 1.54.0) in R (Wilkerson and Hayes, 2010) to perform consistent clustering on the pathway dysregulation profile obtained by the GSVA method. The optimal number of clusters is determined by the cumulative density function (CDF), which plots the corresponding empirical cumulative distribution defined in the range between 0 and 1, and the optimal cluster is determined by calculating the proportional increase in the area under the CDF curve number. When any further increase in the number of clusters (K) does not result in a corresponding significant increase in the area of the CDF, the number of clusters is determined.

Our consistent clustering methods included pam, kmdist, and hc, and clustering measures included Pearson, Spearman, maximum, Minkowski, Manhattan, binary, Canberra, and Euclidean methods. Using each method and each measurement to cluster cancer samples, the number of categories ranged from 2 to 8, reps = 50, pItem = 0.8, and pFeature = 1, and a total of 112 clustering results were obtained.

Then, under the same clustering number, we compared the overlapping rate among these clustering results using the Wilcoxon rank-sum test.

### Kaplan–Meier and log-rank tests

We used the R packages “survival” (version 3.2–7) and “survminer” (version 0.4.9) to calculate the survival difference among subtypes; log rank *p* < 0.05 represents a significant difference.

### Identification of differentially expressed genes

Subtype-specific differentially expressed genes were identified (Wilcoxon text *p* < 0.05; |log2FC| > 1) by using the R packages “edgeR” (version 3.32.1) (Robinson et al., 2010) and “limma” (3.46.0).

### Cox proportional hazards regression model

We performed a univariate Cox regression analysis on 65 differentially expressed genes among subtypes (*p* < 0.01), and then, 56 genes that correlated with the prognosis were identified (*p* < 0.01). Then, the multivariate Cox proportional analysis was performed, and 30 genes were regarded as candidate prognostic genes. To identify independent predictors that significantly contributed to OS or RFS, we constructed a risk model based on these 30 genes and calculated the risk score of each patient using the predict() function in the survival package.

RiskScore=∑βi×Xi,

where βi represents the risk regression coefficient of the multiple Cox analysis corresponding to each gene, and Xi represents the gene expression value. The samples were divided into high- and low-risk groups based on the median value of the risk score for subsequent analysis.

### Random forest and support vector machine to construct the subtype and prognosis classifiers

We used random forest and support vector machine (SVM) methods to construct the subtype and prognostic classifiers by using the R packages “randomForest” (version 4.6–14) and “e1071” (version 1.7–6) in the training data set and then used the test data set to test the performance of the classifiers. In the random forest method, we set the cutoff to 0.5 so that every tree “votes”. Next, we used the importance function to calculate the accuracy of the model variables and the gini coefficient to judge the importance of the variables. The mean value of the gini index change was used as a measure of the importance of the variables, and all features were sorted according to their importance.

### Fisher’s exact test

We used Fisher’s exact test (Blevins and McDonald, 1985) to calculate the difference in copy number amplification and deletion between each two subtypes (*p* < 0.01; FDR > 2).

### Integrative genomics viewer to visualize ATAC-seq data

IGV (Integrative Genomics Viewer) (Thorvaldsdottir et al., 2013) is a tool that can visualize sequencing data on a local computer. For the ATAC-seq bw file of each sample, IGV (version 2.7.0) was used to visualize the chromatin accessibility at the genome position of each subtype.

### Weighted gene co-expression network to identify the methylation sites

The R package WGCNA (version 1.70–3) (Langfelder and Horvath, 2008) was used to build a weighted gene co-expression network. First, the soft threshold *β* was screened to ensure that the constructed network was more in line with the characteristics of the scale-free network. Next, the one-step method was used to construct the network, and gene clustering was performed based on TOM. Then, we used the hierarchical clustering tree to display each module and obtained the correlation between the modules. The correlations between characteristic methylation sites and clinical phenotypes were assessed by Pearson’s correlation analysis, and the correlation coefficients between modules and clinical phenotypes were used to select modules for a downstream analysis.

### MCP to calculate the cell infiltration fraction

We used the R package MCPcounter (version 1.2.0) (https://github.com/ebecht/MCPcounter) to calculate the infiltration fraction of T cells, CD8 T cells, cytotoxic lymphocytes, NK cells, B cells, monocytes, bone marrow dendrites, neutrophils, endothelial cells, and fibroblasts based on gene expression data in GDC.

## Discussion

Cancer subtypes have broad prospects in understanding cancer and personalized treatment (Cao et al., 2018; Guo et al., 2019). However, many studies so far have been based on single cancer. Analyzing from a pan-cancer perspective can identify the differences and commonalities across different cancer types. Signaling pathways change in different combinations among cancers, and there are complex interactions between pathways (Jackstadt et al., 2019; Li et al., 2020). But the extent, mechanism, and co-occurrence of these pathway changes varied across tumors and tumor types.

We divided patients of TCGA 32 cancer types into four molecular subtypes; although our project covered most tissues and organ systems, some tumor types including most hematologic cancers were not included. Also, we did not combine the known molecular subtypes of certain cancer types for our analysis. Then, the biomarkers among subtypes were identified at the multi-omics levels. A multi-omics analysis is of great significance for revealing cancer development, treatment resistance, and recurrence risk, and it is the key to advancing precision medicine in clinical practice. However, we did not conduct further and deeper mining of multi-omics biomarkers we found. In addition, drug sensitivity requires clinical evaluation; then well-designed clinical trials are expected to test the possibility of translating our results to clinical practice in the future.

In conclusion, our study provided a new perspective to understand the relationship of the dysregulation of oncogenic signaling pathways and cancers and identified potential prognostic biomarkers from multiple omics data, and it further might have implications for clinical applications in the future.

## Conclusion

Here, based on gene set variation analysis (GSVA), we constructed a pathway dysregulation landscape and identified four subtypes based on oncogenic signaling pathways in pan-caner, which may provide an increased understanding of the common molecular mechanisms driven by oncogenic signaling pathways underlying the pathogenesis of the malignancy. These four subtypes showed distinct patient prognosis, cancer type distributions, transcriptional changes, chromatin accessibility, genomic alterations, methylation degree, and tumor microenvironment characteristics. Several signature sets were identified by integrating multi-omics profiles, which were used to construct a subtype classifier and a prognosis prediction model. Overall, our analysis demonstrates that the molecular heterogeneity of oncogenic signaling pathways, improves the understanding of the mechanisms of oncogenic signaling pathways driving tumor progression, and enables the development of personalized therapies targeting unique tumor oncogenic signaling pathway dysregulation profiles.

## Data Availability

The original contributions presented in the study are included in the article/Supplementary Material; further inquiries can be directed to the corresponding authors.

## Supplementary material

Supplementary Figure S1
**(A)** CDF curve with k = 2–8 using the kmdist–Spearman method. **(B)** Area under the CDF curve for the kmdist–Spearman method, k = 2–8. **(C)** Sample cluster distribution using the kmdist–Spearman method, k = 2–8. **(D)** Signaling pathway–based clustering results of TCGA training cohort (*n* = 7518). The heat map shows normalized enrichment scores of the four subtypes.

Supplementary Figure S2
**(A,C)** Forest plot of the multiple Cox regression analysis on the pathological stage for OS **(A)** and DFS**(C)**. **(B,D)** Forest plot of the multiple Cox regression analysis on age and sex for OS **(B)** and DFS **(D)**. The hazard ratios are shown with 95% confidence intervals.

Supplementary Figure S3 Kaplan–Meier survival curves among the four subtypes of 22 cancers.

Supplementary Figure S4
**(A)** Drugs used by more than 50 patients (red). **(B)** Sensitivity of four subtypes of patients to different situations with treatment. (None: sensitive; relapse, transfer, both: insensitive).

Supplementary Figure S5
**(A)** Interactions of genes in the cell cycle pathway and fold change values of four subtypes of genes. **(B)** Interaction of genes in the MYC pathway and the fold change values of the four subtypes of the genes. **(C)** Interactions of genes in the NOTCH pathway and fold change values of four subtypes of genes.

Supplementary Figure S6 Interactions of genes in the RTK/RAS pathway and fold change values of four subtypes of genes.

Supplementary Figure S7 Interactions of genes in the WNT pathway and fold change values of four subtypes of genes.

Supplementary FIGURE S8 Infiltration of immune cells in MCP and TIMER in four subtypes.

Supplementary Table S1 TCGA dataset.

Supplementary Table S2 Differentially expressed genes across the four subtypes.

Supplementary Table S3 Differentially methylated sites.
