# Supplementary figures and images for: Oncogenic signaling pathway dysregulation landscape reveals the role of pathways at multiple omics levels in pan-cancer
Source: Front Genet. 2022 Aug 17;13:916400. doi: 10.3389/fgene.2022.916400 (PMC9428557; doi:10.3389/fgene.2022.916400)

A

## Cell Cycle pathway

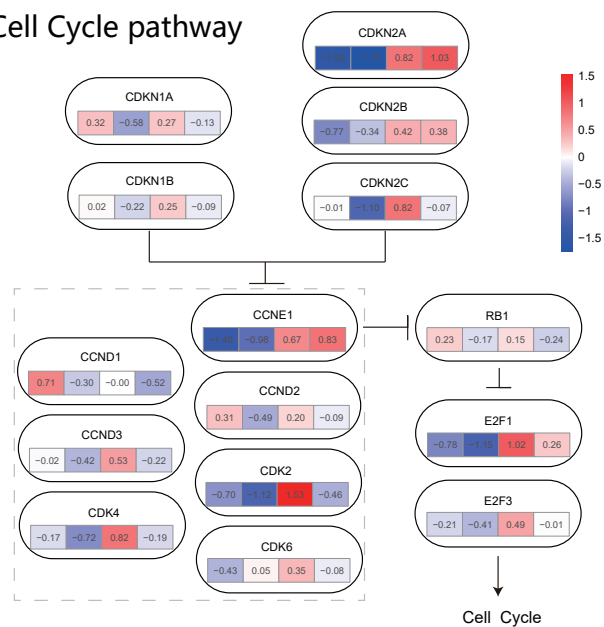

B

## MYC pathway

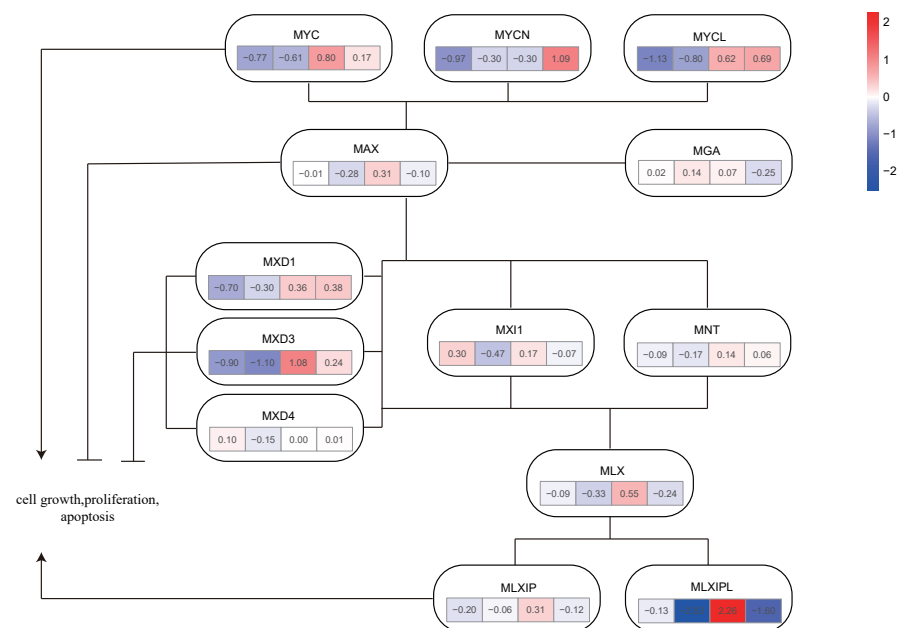

C

## NOTCH pathway

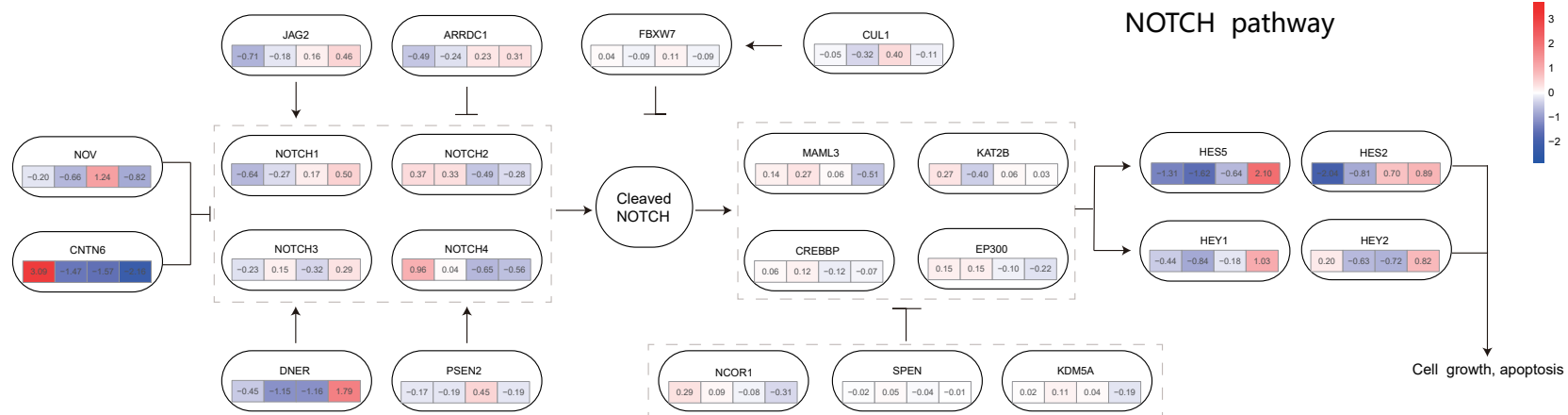

Supplement: Supplementary file 1 [file Image5.PDF]

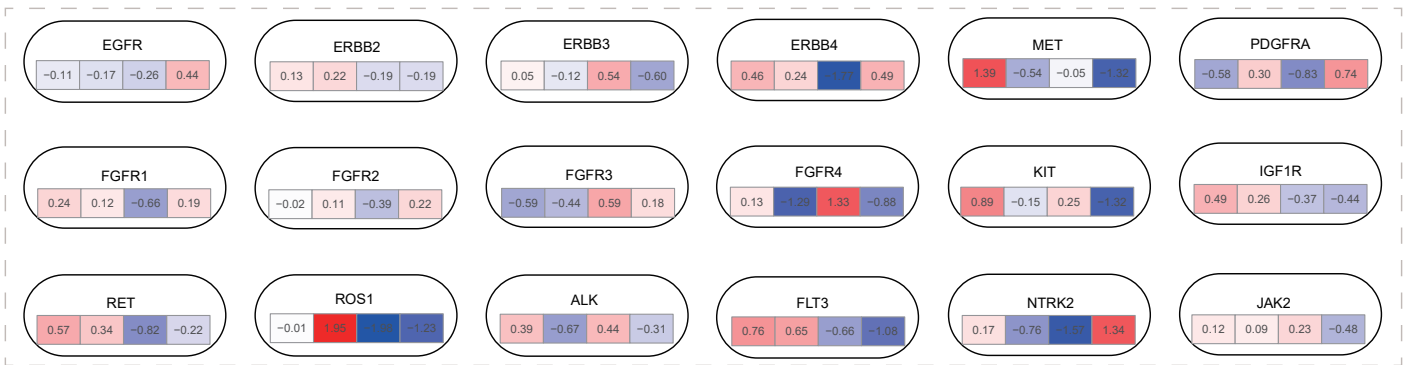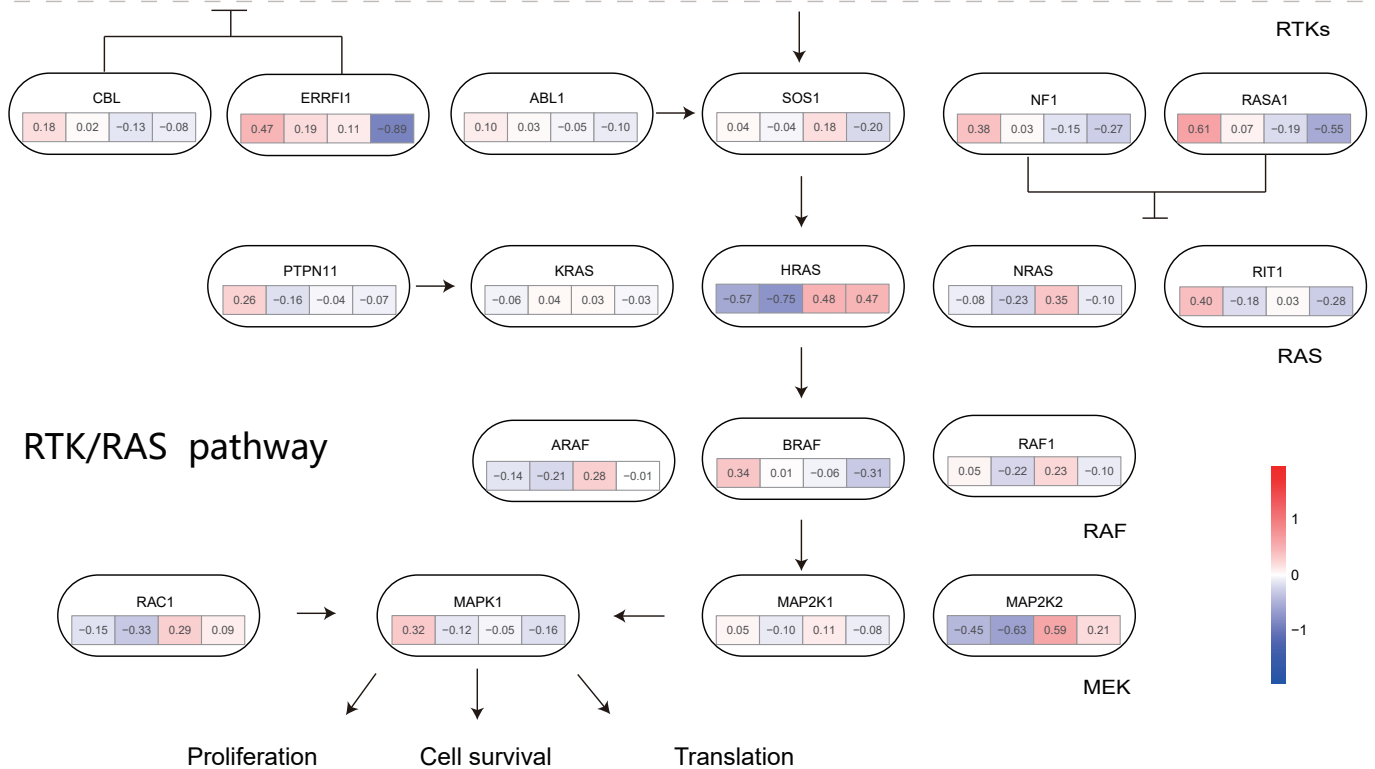

Supplement: Supplementary file 4 [file Image6.PDF]

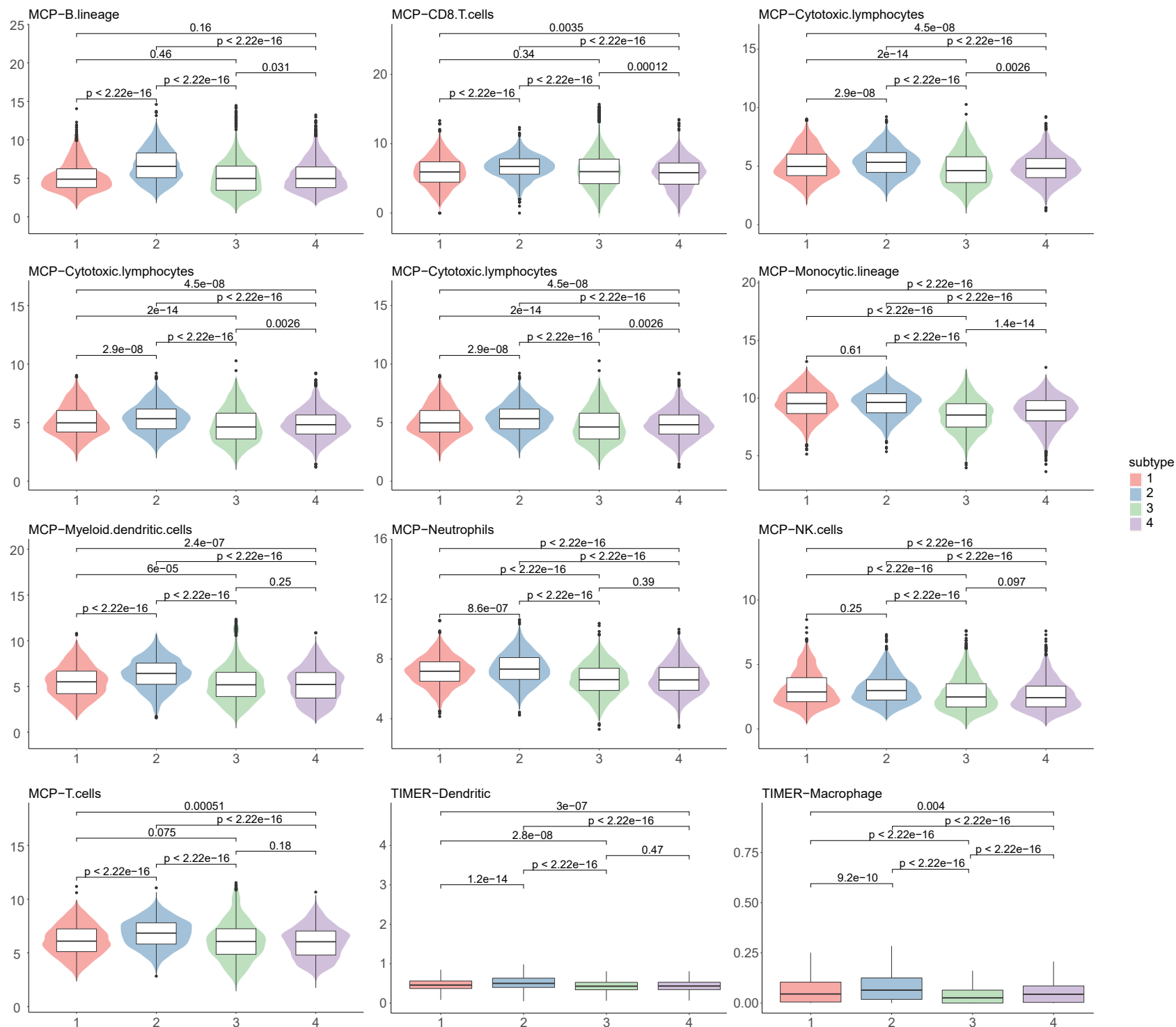

Supplement: Supplementary file 5 [file Image8.PDF]

A

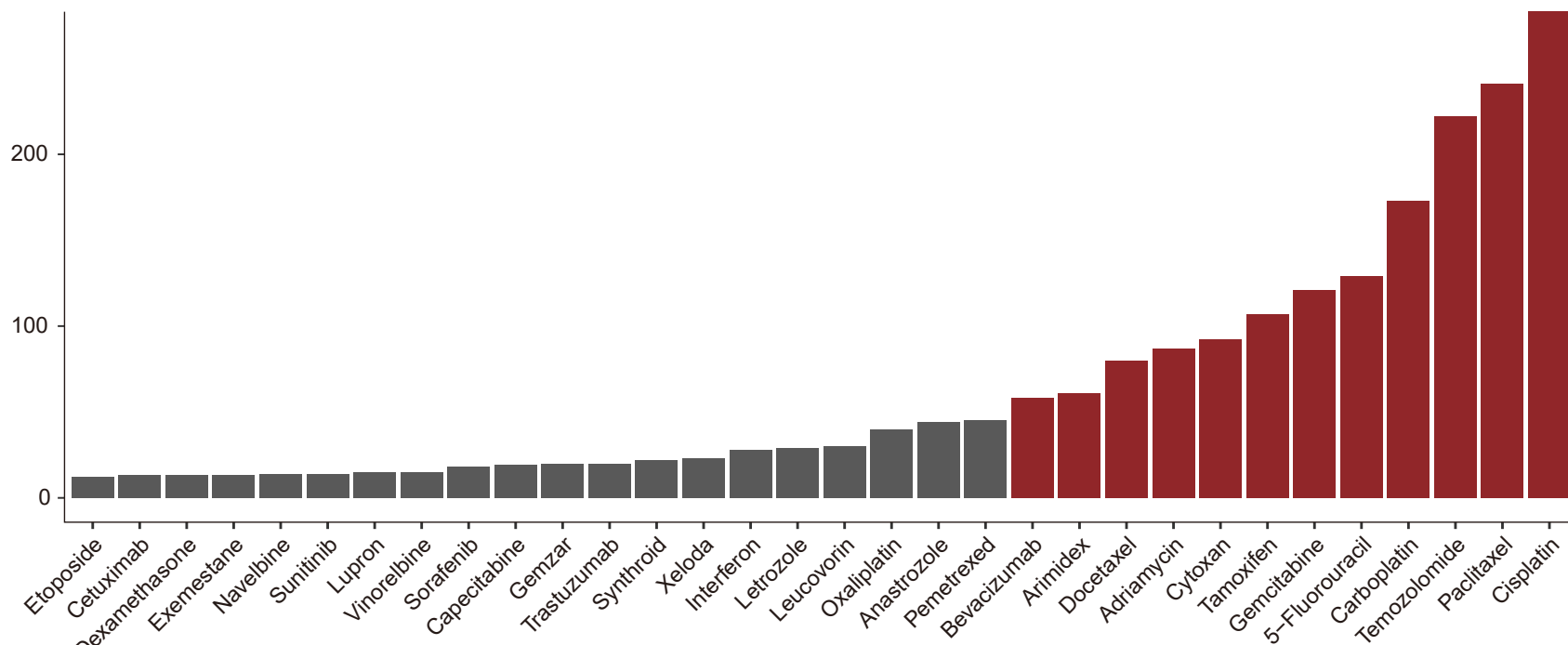

B

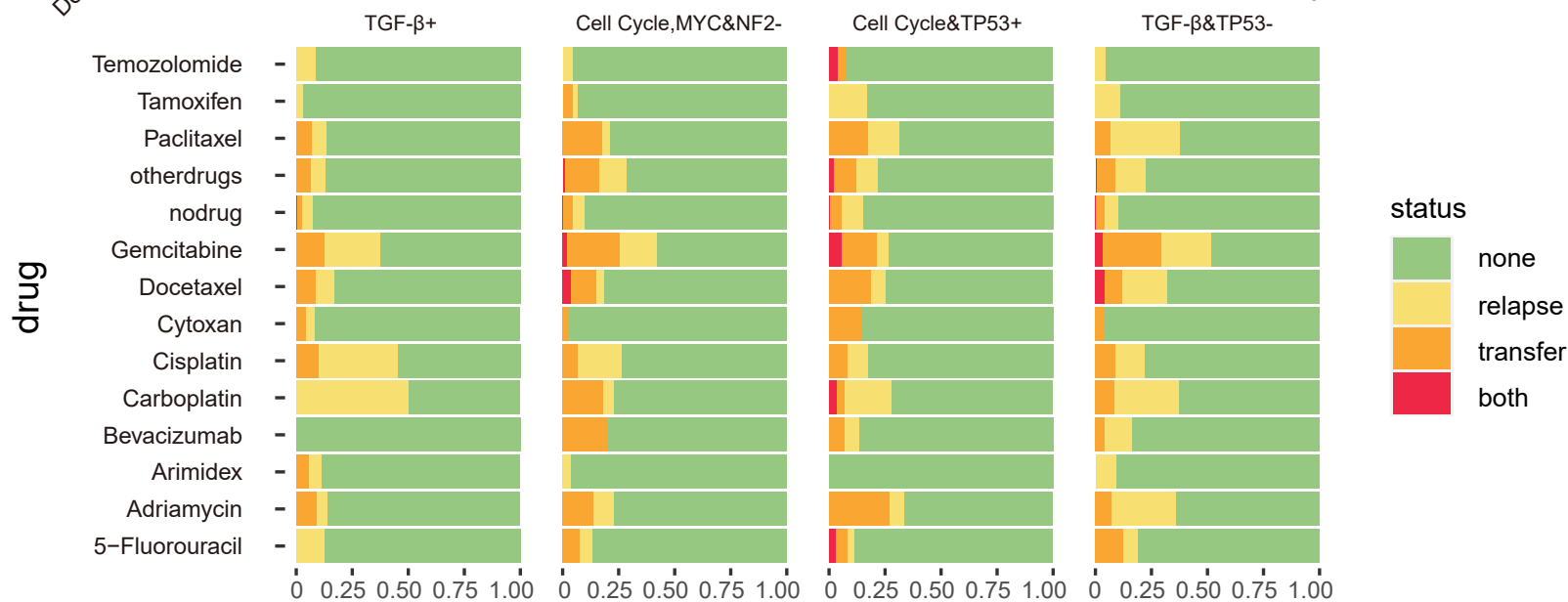

Supplement: Supplementary file 7 [file Image4.PDF]

A

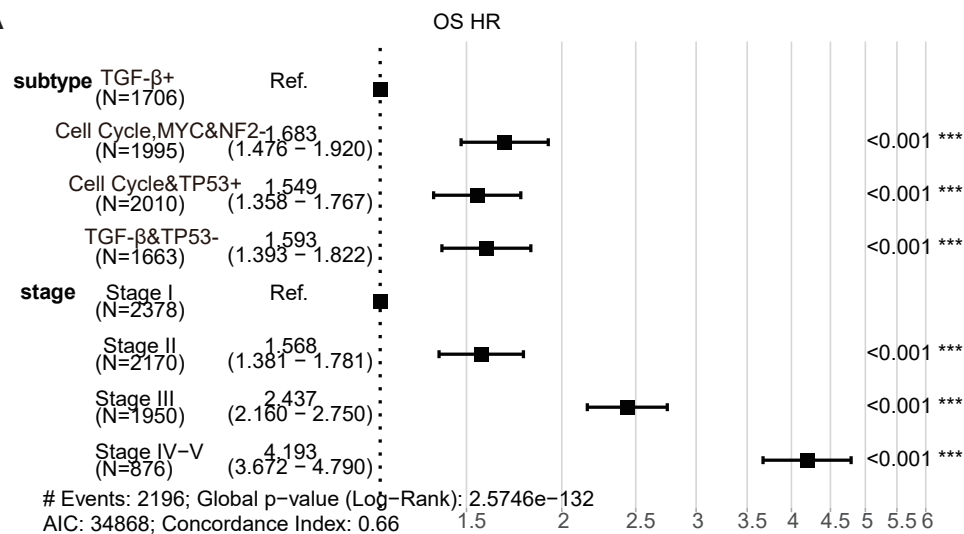

B

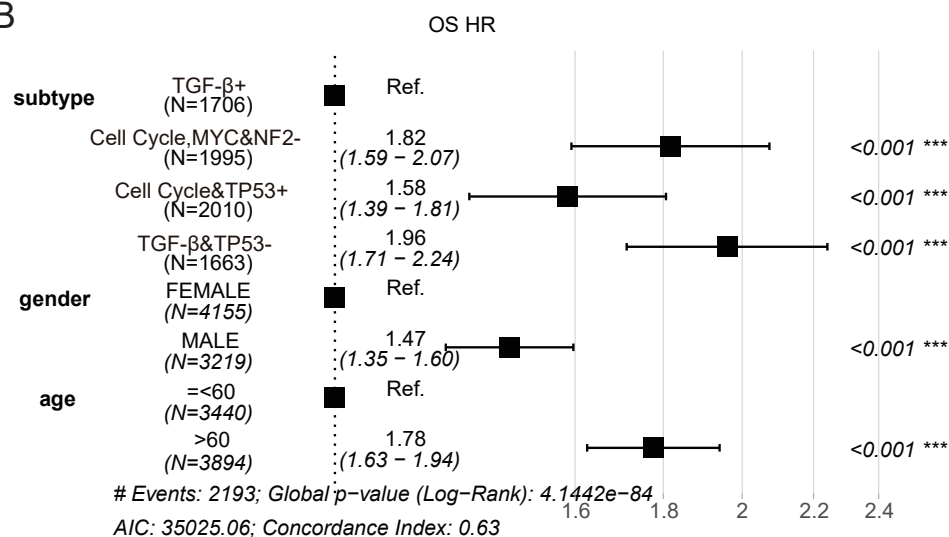

C

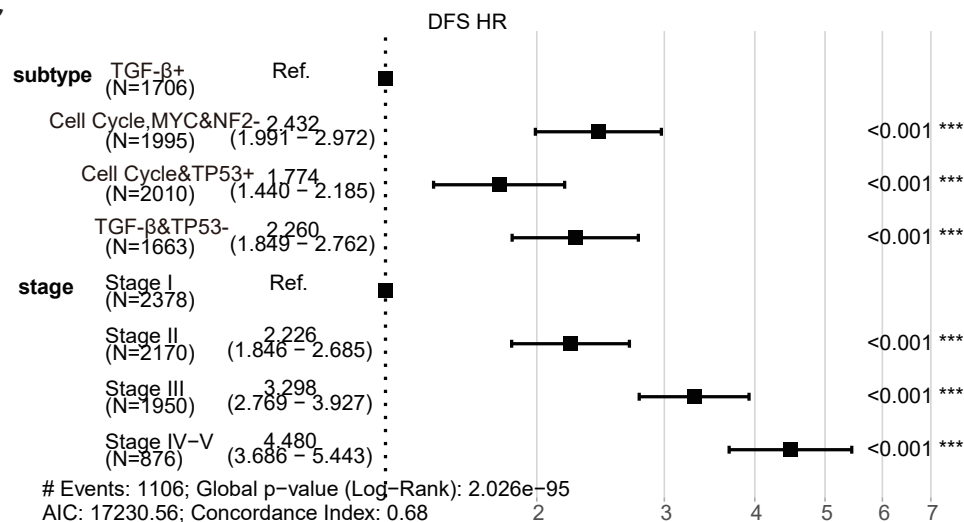

D

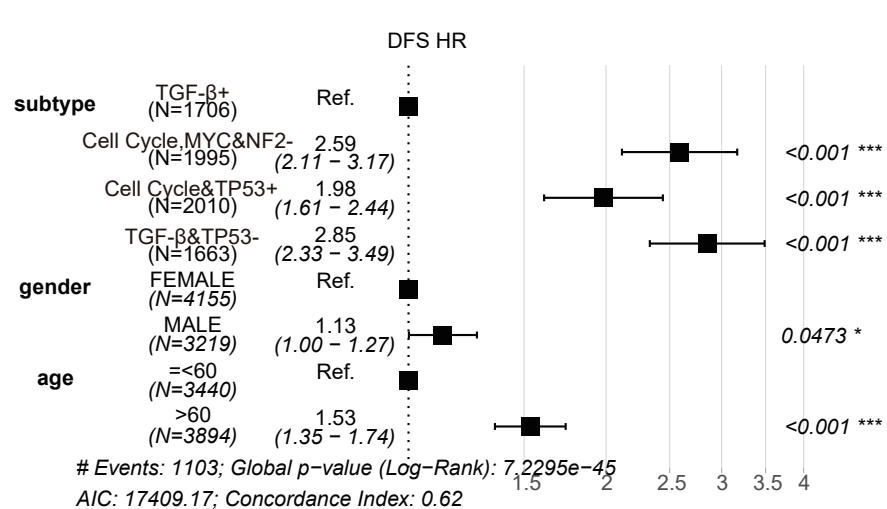

Supplement: Supplementary file 8 [file Image2.PDF]

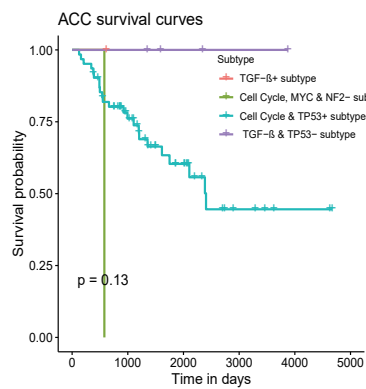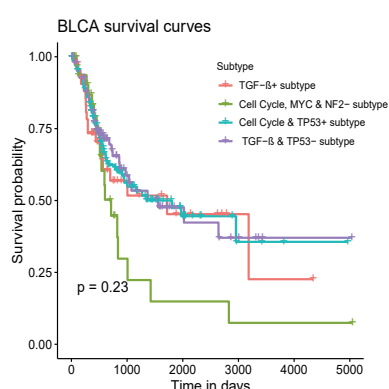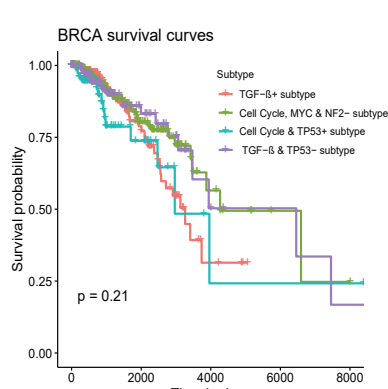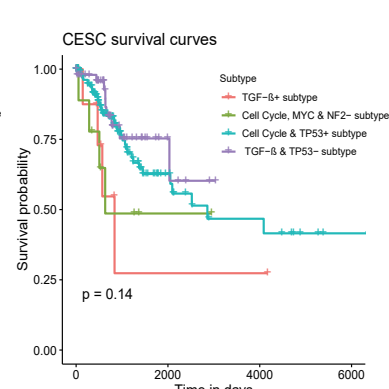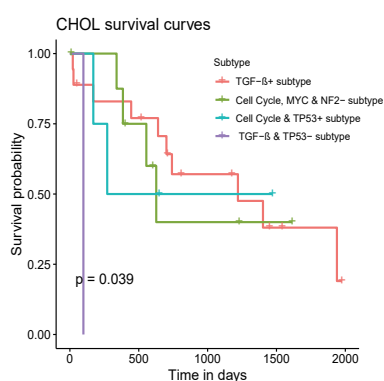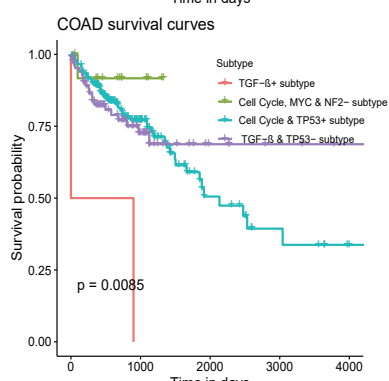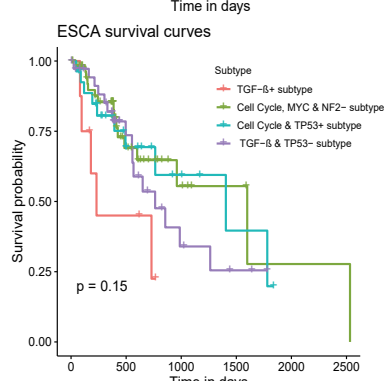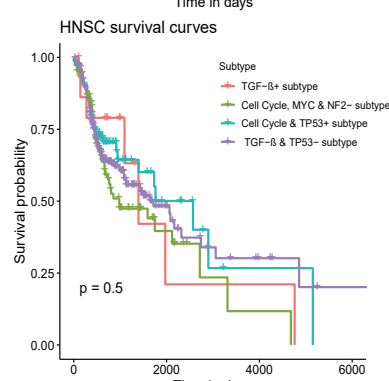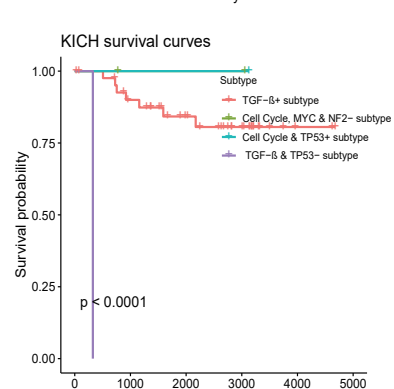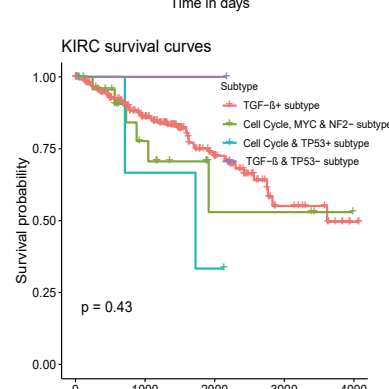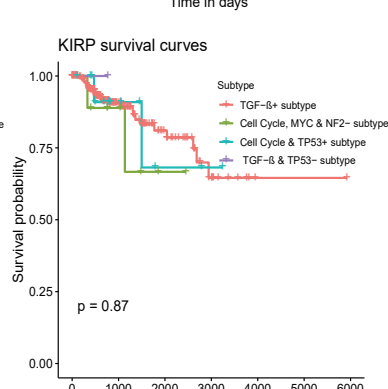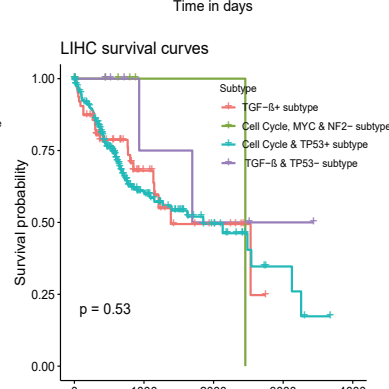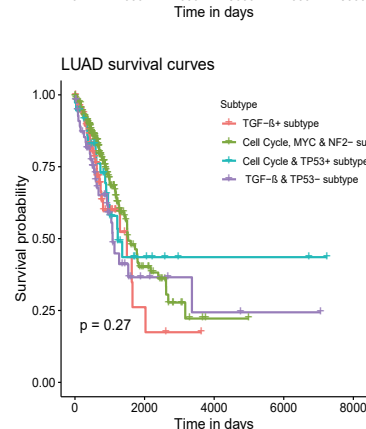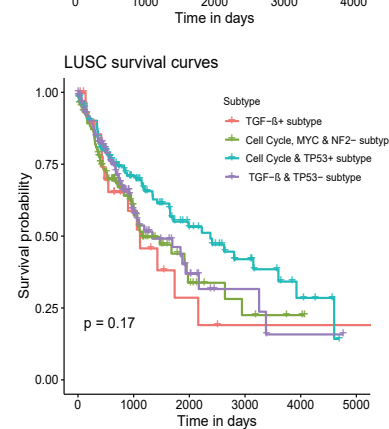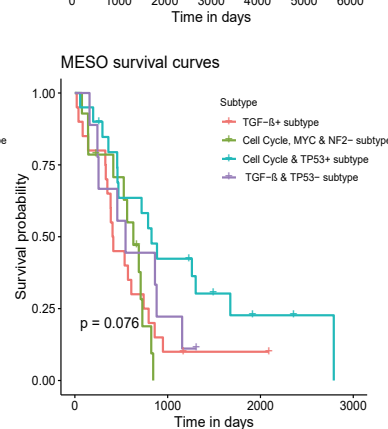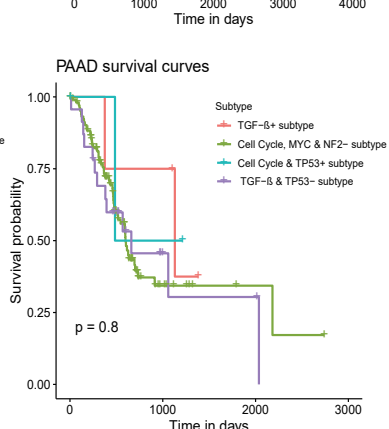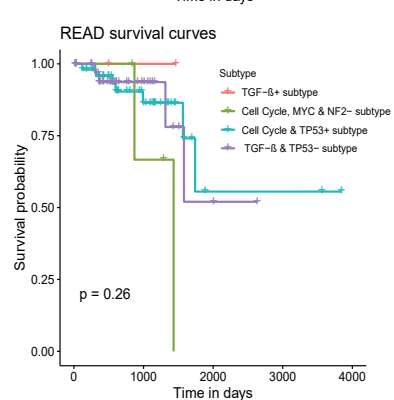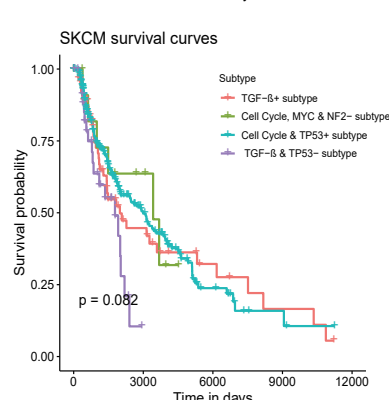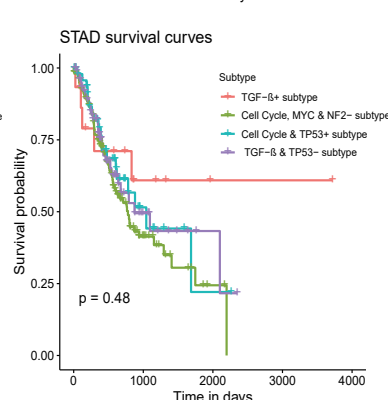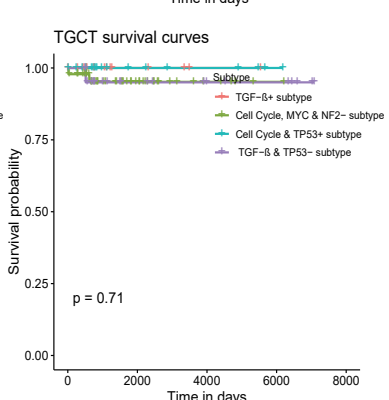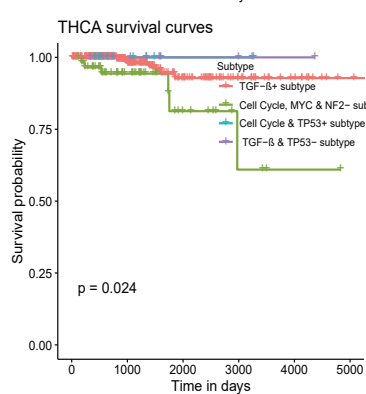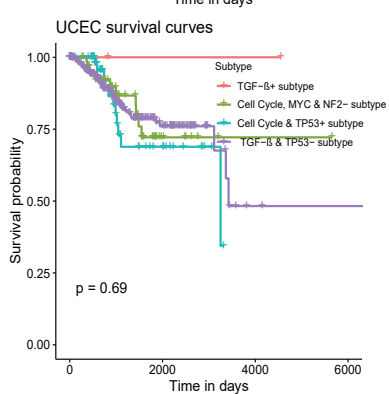

Supplement: Supplementary file 9 [file Image3.PDF]

# WNT pathway

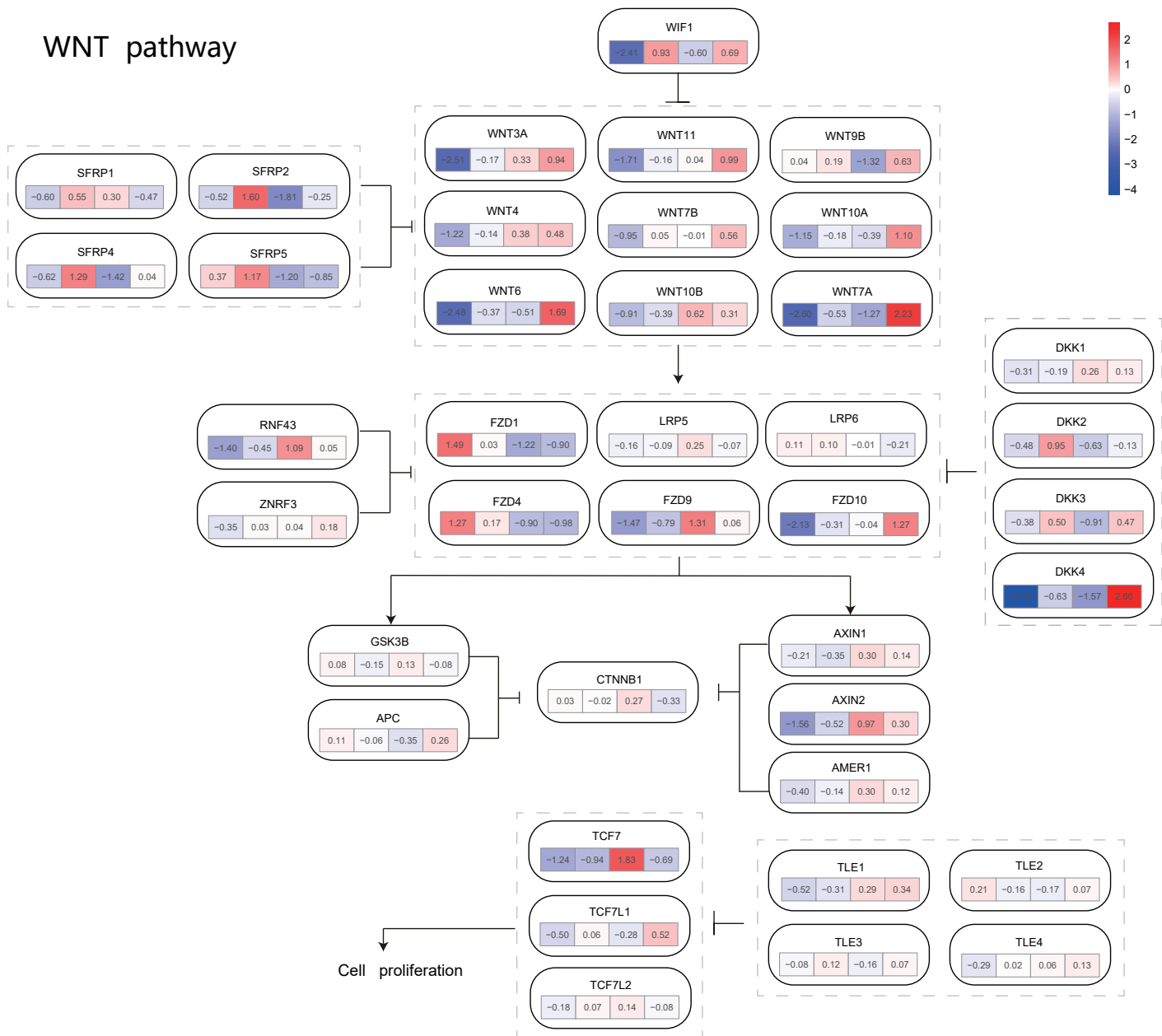

Supplement: Supplementary file 10 [file Image7.PDF]

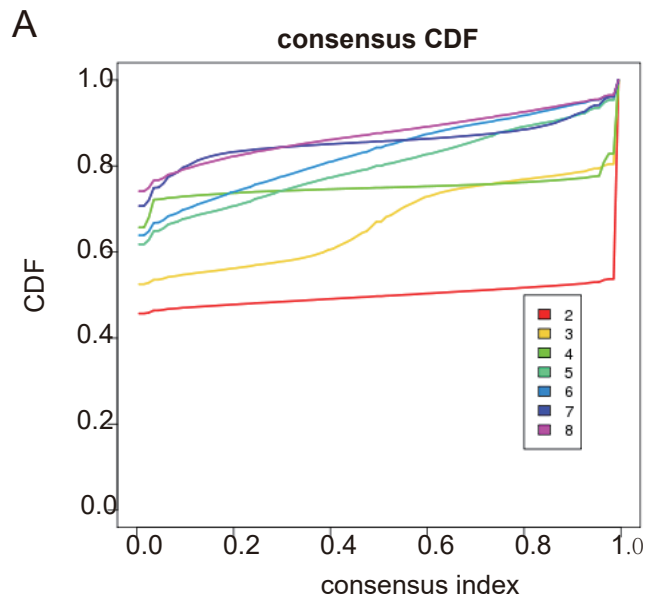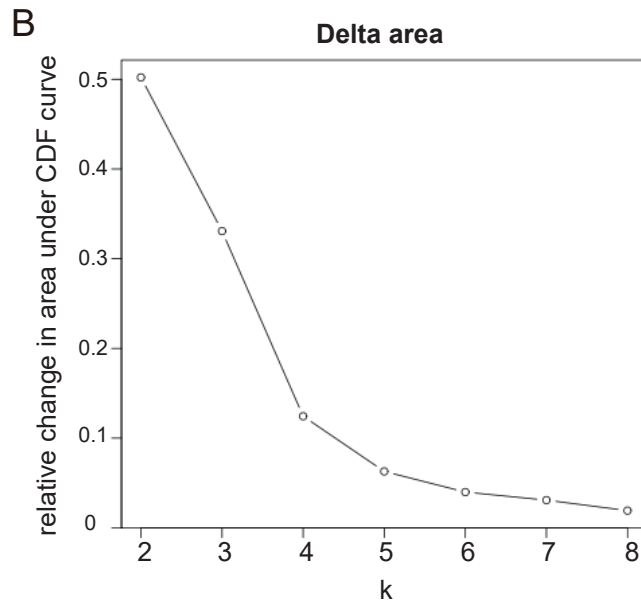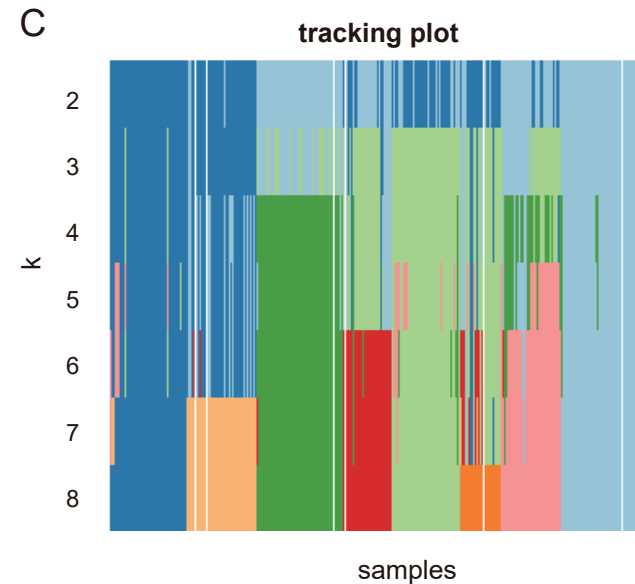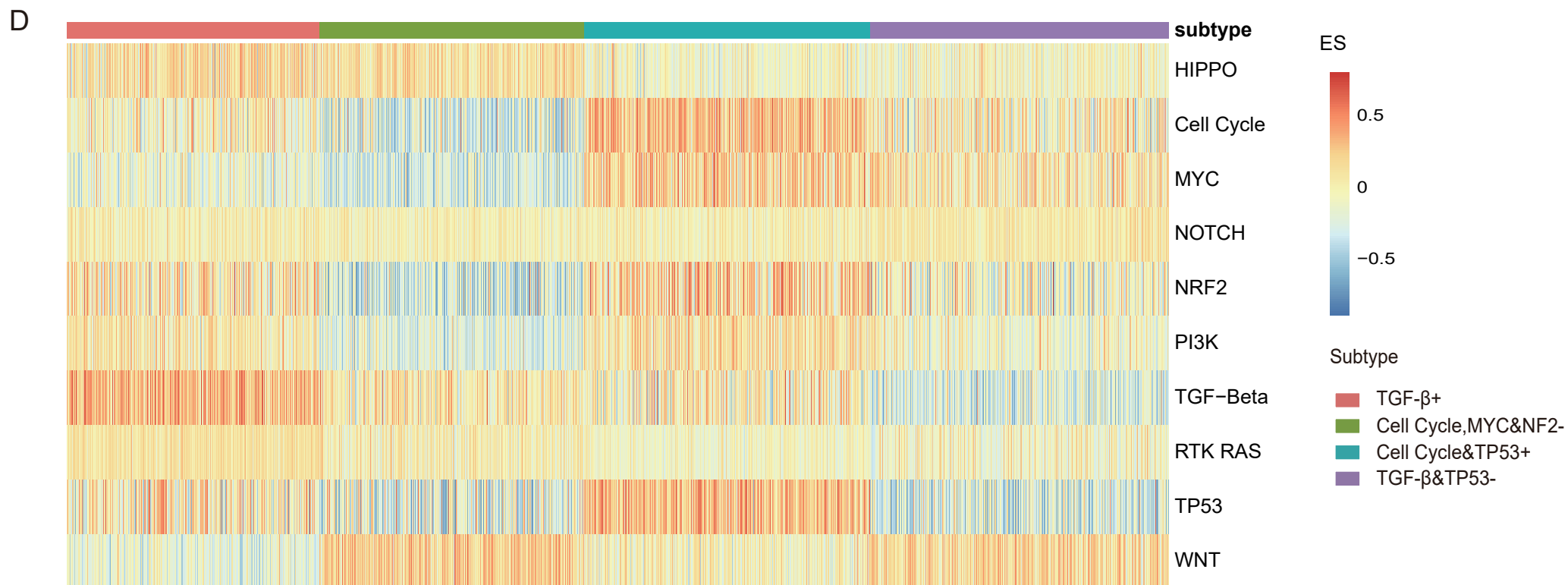

Supplement: Supplementary file 11 [file Image1.PDF]
