# Supplementary material for: Oncogenic signaling pathway dysregulation landscape reveals the role of pathways at multiple omics levels in pan-cancer
Source: Front Genet. 2022 Aug 17;13:916400. doi: 10.3389/fgene.2022.916400 (PMC9428557; doi:10.3389/fgene.2022.916400)
Supplement: Supplementary file 3 [file Table1.docx]

Table s1 TCGA expression dataset

| Cancer types | Abbreviation | Sample numbers |
| --- | --- | --- |
| adrenocortical carcinoma | ACC | 79 |
| bladder urothelial carcinoma | BLCA | 408 |
| breast invasive carcinoma | BRCA | 981 |
| cervical squamous cell carcinoma and endocervical adenocarcinoma | CESC | 286 |
| cholangiocarcinoma | CHOL | 36 |
| colon adenocarcinoma | COAD | 396 |
| lymphoid neoplasm diffuse large B-cell lymphoma | DLBC | 37 |
| esophageal carcinoma | ESCA | 163 |
| glioblastoma multiforme | GBM | 156 |
| head and neck squamous cell carcinoma | HNSC | 495 |
| kidney chromophobe | KICH | 66 |
| kidney renal clear cell carcinoma | KIRC | 332 |
| kidney renal papillary cell carcinoma | KIRP | 279 |
| lower grade glioma | LGG | 504 |
| liver hepatocellular carcinoma | LIHC | 359 |
| lung adenocarcinoma | LUAD | 511 |
| lung squamous cell carcinoma | LUSC | 489 |
| mesothelioma | MESO | 81 |
| ovarian serous cystadenocarcinoma | OV | 274 |
| pancreatic adenocarcinoma | PAAD | 172 |
| pheochromocytoma and paraganglioma | PCPG | 179 |
| prostate adenocarcinoma | PRAD | 492 |
| rectum adenocarcinoma | READ | 133 |
| sarcoma | SARC | 235 |
| skin cutaneous melanoma | SKCM | 466 |
| stomach adenocarcinoma | STAD | 377 |
| testicular germ cell tumors | TGCT | 145 |
| thyroid carcinoma | THCA | 487 |
| thymoma | THYM | 118 |
| uterine corpus endometrial carcinoma | UCEC | 526 |
| uterine carcinosarcoma | UCS | 56 |
| uveal melanoma | UVM | 80 |
